# Phenotype and regulation of immunosuppressive Vδ2-expressing γδ T cells

**DOI:** 10.1007/s00018-013-1467-1

**Published:** 2013-10-04

**Authors:** Christian Peters, Hans-Heinrich Oberg, Dieter Kabelitz, Daniela Wesch

**Affiliations:** Institute of Immunology, Christian-Albrechts University of Kiel, Arnold-Heller Strasse 3, Haus 17, 24105 Kiel, Germany

**Keywords:** Immunosuppressive Vδ2 γδ T cells, Helios, FoxP3, CTLA-4, CD86, Toll-like receptor 2 ligands, Phosphorylation

## Abstract

The proliferation and interleukin-2 production of CD4^+^CD25^−^ αβ T cells were inhibited in a cell-contact manner by Vδ2 γδ T cells. The transcription factor Helios was constitutively expressed in about one-third of circulating γδ T cells and was upregulated by CD28-signaling. Our data suggest that Helios could serve as a marker for differential activation status rather than for regulatory T cells (Treg). Our findings also indicate that the interaction of CD86 on activated Vδ2 T cells and cytotoxic T lymphocyte-associated antigen-4 (CTLA-4) on activated αβ T cells mediated the suppression because the suppressive effect was abolished by blocking the CD86:CTLA-4 interaction. Pre-treatment of Vδ2 T cells with Toll-like receptor 2 ligands enhanced phosphorylation of MAPKs, Akt, and NF-κB and partially abrogated the suppressive capacity, whereas on co-cultured responder T cells inhibitory molecules were downregulated and Akt and NF-κB phosphorylation was restored. Our results suggest that the regulation of αβ T cell proliferation by activated Vδ2 T cells might contribute to fine-tuning of αβ T cell responses.

**Electronic supplementary material:**

The online version of this article (doi:10.1007/s00018-013-1467-1) contains supplementary material, which is available to authorized users.

## Introduction

Vγ9Vδ2 γδ T cells are the main subset of the 1–10 % of γδ T lymphocytes in the human blood and upon activation display characteristics of both the innate and the adaptive immune system. The innate immune properties of activated Vδ2 γδ T cells include phagocytosis/trogocytosis, cross-presentation of soluble antigen, antibody-dependent cell-mediated and natural killer (NK)-receptor-mediated cytotoxicity [1–6]. In contrast, characteristics of the adaptive immune system imply the necessity of two signals [T cell receptor (TCR) and co-stimulatory molecules] to induce proliferation or the need for TCR stimulation to enhance Toll-like receptor (TLR) ligand-mediated cytokine production [7–9]. Vδ2 γδ T cells recognize phosphorylated intermediates [phosphoantigens (PAg)] of the non-mevalonate pathway in prokaryotes or the accumulation of PAg in the dysregulated mevalonate pathway in eukaryotes [10]. They also produce large amounts of interferon gamma (IFN-γ) and tumor necrosis factor alpha (TNF-α) and high levels of CCL5 (RANTES) and CCL3 (MIP-1α) as well as cytolytic effectors, such as granzyme B [11]. Initially activated Vδ2 γδ T cells are poor producers of interleukin-2 (IL-2). For proliferation, Vδ2 γδ T cells require either the endogenous production of IL-2 by antigen-stimulated CD4 T cells or an exogenous supply of IL-2 [12]. Vδ2 γδ T cells comprise distinct populations distinguishable on the basis of surface markers, trafficking properties, and effector functions. CD45RA/CD27-positive naive γδ T cells (T_naive_) as well as CD45RA-negative and CD27-positive central memory T cells (T_CM_) have the ability to home to secondary lymphoid organs and they lack immediate effector functions. In comparison, CD45RA/CD27-negative effector-memory T cells (T_EM_) and CD45RA-positive and CD27-negative terminally differentiated effector memory T cells (T_EMRA_) have the ability to home to sites of inflammation where they display immediate effector functions, such as cytokine production and cytotoxicity [13, 14].

In general, γδ T lymphocytes exhibit cross-regulatory interactions with other cells of the immune system, and they have been shown to fulfill both co-stimulatory and suppressive functions [15–17].

Thymic-derived natural regulatory T cells (nTreg) regulate self-tolerance and prevent autoimmunity [18]. They highly express CD25, cytotoxic T lymphocyte-associated antigen-4 (CTLA-4/CD152) and the transcription factors forkhead box (Fox) P3 and Helios [19–21]. FoxP3 acts as a transcriptional repressor of IL-2 and IFN-γ genes, which results in the suppressive capacity of Treg on the proliferation of other cells [22]. In contrast to mice, FoxP3 expression in humans does not necessarily confer regulatory function because transient FoxP3 expression can be induced after activation in peripheral non-Treg detected with FoxP3 antibody PCH101 [21, 23, 24]. FoxP3 expression detected with anti-FoxP3 monoclonal antibody (mAb) 259D is induced after γδ T cell activation in the presence of transforming growth factor beta (TGF-β) and IL-15, a process which seems to be linked to suppressive function [24–26]. Regarding FoxP3 expression stained with 259D mAb, no data are available on Vδ2 γδ T cells activated with PAg- or anti-CD3/anti-CD28 mAb in the absence of TGF-β and IL-15. The transcription factor Helios is a member of the Ikaros family of DNA-binding zinc finger regulators which includes Ikaros, Aiolos, Eos, and Pegasus. Helios is highly expressed in the earliest stage of T cell development [27]. In one study Helios was reported to distinguish thymic-derived Treg from peripherally induced FoxP3^+^ Treg [20], but more recent studies have shown that Helios can be up-regulated after activation in FoxP3^+^ iTreg [adaptive FoxP3^+^CD4^+^ Treg] as well as in human responder TCRαβ^+^ FoxP3^−^ T cells [28–30]. Overexpression of dominant-negative or full-length Helios isoforms alters the differentiation and activation of αβ T cells and also induces increased numbers of NK cells and γδ T cells in peripheral lymphoid organs [31]. In contrast, Cai et al. [32] reported that inactivation of the Helios gene by homologous recombination does not impair the effector function and differentiation of Treg and αβ-, γδ-, and NKT cells, suggesting that the Helios function can be compensated by other Ikaros family members.

Inhibitory receptors such as CTLA-4 and programmed cell death (PD)-1 play a key role in immune regulation [33]. CTLA-4 is expressed on Treg and upregulated upon TCR activation in non-Treg. The higher affinity and avidity of CTLA-4 for CD80 and CD86 expressed on antigen-presenting cells (APC) as compared to constitutively expressed co-stimulatory CD28 antagonize early T cell activation (mediated by CD28:CD80/CD86 interaction) and limit an immune response (by means of, for example, decreased IL-2 production) [34, 35]. CD28 signaling results in a phosphoinositide 3 kinase (PI3 K)-dependent phosphorylation of the serine/threonine kinase Akt and an activation of nuclear factor ‘kappa-light-chain-enhancer’ of activated B-cells (NF-κB), thereby influencing cell survival [36]. However, recent publications have discussed a dual function of CD28 and CTLA-4, both of which are expressed on Treg and on non-Treg, suggesting that both molecules are critical regulators of Treg homeostasis and function [37]. PD-1 (CD279) is induced on TCR-activated T cells and delivers co-inhibitory signals upon binding to its ligands (L) PD-L1 (CD274) or PD-L2 (CD273). PD-L1 is expressed on APC as well as on T cells, whereas PD-L2 is inducibly expressed on dendritic cells (DC) and macrophages. Ligation of TCR and PD-1 induces tyrosine phosphorylation of PD-1 and recruitment of SHP-2, which dephosphorylates TCR proximal molecules (e.g., ZAP70, CD3ζ) and attenuates the TCR/CD28 signal [33, 34, 38].

In earlier studies we demonstrated that human Treg treated with TLR2 ligands lose their suppressive capacity, which is based on a restoration of Akt phosphorylation and downregulation of the cdk inhibitor p27^Kip1^ [39, 40]. Recognition of bacterial lipopeptides by TLR1–TLR2 or TLR2–TLR6 heterodimers results in the induction of signaling cascades which in turn activate NF-κB as well as mitogen-activated protein kinases (MAPK) [41, 42].

In the study reported here, we have examined the characteristic features of suppressive Vδ2 γδ T cells and the molecular mechanisms responsible for their suppressive function.

## Materials and methods

### Leukocyte concentrates

The Department of Transfusion Medicine in Kiel, Germany provided leukocyte concentrates from healthy adult blood donors who gave their informed consent. The study was approved by the relevant institutional review boards (code number: D 405/10).

### Isolation of T cell populations and cell cultures

Peripheral blood mononuclear cells (PBMC) were isolated from the leukocyte concentrates by Ficoll-Hypaque (Biochrom, Berlin, Germany) density gradient centrifugation. PBMC were screened for the expression of CD4, CD25^high^, and Vδ2 γδ T cells. PBMC with γδ T cells which consisted of only Vδ2 T cells were used; CD4^+^CD25^−^ responder T cells and CD4^+^CD25^high^ Treg were purified from the PBMC using a magnetic cell separation system (Miltenyi Biotec, Bergisch-Gladbach, Germany). γδ T cells were isolated by positive selection (anti-TCRγ/δ MicroBead Kit; Miltenyi Biotec), and responder T cells by negative selection (CD4^+^ T Cell Isolation Kit II; Miltenyi Biotec) followed by the positive selection of Treg by Dynabeads (Life Technologies, Carlsbad, CA). DETACHaBEAD (Life Technologies) were used to remove magnetic particles from positively selected cells. Optimized separation conditions (e.g., usage of two consecutive MACS columns) revealed that the isolated T cell subpopulations had a purity of >98 %. To avoid (pre)activation, purified T cells were cultured in serum-free X-VIVO 15 medium (Lonza, Cologne, Germany) for 22 h at 37 °C in an incubator. Our previously published APC-free suppression assay was applied as the read-out system for cell proliferation [43]. In brief, 10^4^ purified responder T cells, 10^4^ purified autologous γδ T cells or Treg or the co-culture of responder T cells with Treg or γδ T cells were stimulated with Activation/Expander T cell beads (A/E beads; Miltenyi Biotec) as a TCR stimulus in 96-well round-bottom plates. The A/E beads were coated with 10 μg/mL anti-CD3, 10 μg/mL anti-CD28, and 0.5 μg/mL anti-CD2 mAb. Alternatively, where indicated, cells were stimulated with 2 μg/mL coated anti-CD3 mAb (100 μL) or 1 μg/mL soluble anti-CD28 mAb. To investigate the interaction of regulatory molecules between responder and γδ T cells, wells had been additionally pre-coated with of one of the following antibodies (each at 5 μg/mL): anti-CD80 (clone 37711 from R&D Systems, Wiesbaden, Germany), anti-CTLA-4 (clone L3D10), anti-CD86 (clone IT2.2), anti-PD-1 (clone EH12.2H7), anti-PD-L1 (clone 29E.2A3) mAbs and the appropriate isotype controls (all from Biolegend, San Diego, CA). In γδ T cell solo-culture, 50 U/mL IL-2 was added to ensure the proliferation of γδ T cells, which are poor IL-2 producers. The modulation of suppression by TLR2 ligands (TLR2L) was investigated after a 22 h pre-incubation of γδ T cells with a mixture of previously titrated lipopeptides [Pam_2_CSK4 (2 μg/mL), FSL-1 (1 μg/mL), and Pam_3_CSK4 (2 μg/mL); TLR2-L-mix; InvivoGen, Toulouse, France) followed by a washing step.

### Cell proliferation assay

Proliferation was determined by the uptake of tritiated thymidine (^3^H-TdR) during the last 16 h of a 2- to 5-day culture period using a Wallac 1450 Microbeta Trilux counter (Perkin Elmer, Rodgau-Jügesheim, Germany). Results are expressed as mean counts per minute (cpm) ± standard deviation (SD) of triplicate cultures.

The absolute cell number of viable responder or γδ T cells was measured with a flow cytometric method termed the standard cell dilution assay (SCDA) after 6–8 days of culture [44]. Briefly, co-cultured responder and γδ T cells from 96-well round-bottom plates were washed and stained with fluorescein isothiocyanate (FITC)-labeled anti-CD4 mAbs (BD Biosciences, San Jose, CA). After one washing step, cells were resuspended in sample buffer containing a defined number of allophycocyanin-labeled fixed standard cells and 0.2 μg/mL propidium iodide (PI). The standard cells were purified T cells that had been stained with allophycocyanin-labeled anti-HLA class I mAb w6/32 and anti-TCRαβ mAb BMA031 and then fixed in 1 % paraformaldehyde. The analysis on a flow cytometer enabled simultaneous measurement of the expansion of viable CD4 T cells (FITC^+^PI^−^allophycocyanin^−^), viable γδ T cells (FITC^−^PI^−^allophycocyanin^−^), and standard cells (FITC^−^PI^+^allophycocyanin^+^). Based on the known number of standard cells, we could then determine the absolute number of viable CD4^+^ responder T cells and γδ T cells in a given microculture well as described previously [44].

### Establishment of T cell lines

Positively isolated γδ T cells were cultured in RPMI 1640 medium supplemented with 2 mmol/L l-glutamine, 25 mmol/L HEPES, antibiotics, and 10 % fetal calf serum with the following supplements: (1) 300 nmol/L of phosphoantigen bromohydrin pyrophosphate (BrHPP; kindly provided by Innate Pharma, Marseille, France) plus 10 U/mL IL-2; (2) BrHPP plus IL-2, 1.7 ng/mL TGF-β, and 10 ng/mL IL-15; (3) A/E beads plus IL-2; (4) A/E beads plus IL-2, TGF-β, and IL-15. The cytokines were added once again on day 4, 8 and 12 after initial stimulation, and cells were split during the 16-day culture period. The purity of the expanded γδ T cells was >95 % 16 days after culture. Samples of 10^4^ or 5 × 10^4^ of the short-term cultured γδ T cell lines were co-cultured with 10^4^ or 5 × 10^4^ autologous responder T cells, respectively, and re-stimulated with A/E beads or BrHPP with a mixture of *Staphylococcus aureus* enterotoxins A, B, C, D, and E (Serva, Heidelberg, Germany), 40 Gray autologous irradiated PBMC.

### Flow cytometry and optical analysis

The following mAb were used for intracellular staining: Helios, FoxP3 (clones PCH101 and 259D), and the appropriate isotype controls [e-Bioscience (San Diego, CA) and BioLegend (San Diego, CA)]. PCH101 targets the* N*-terminal region of a 431-amino acid protein, whereas 259D recognizes FoxP3 epitopes in the *N*-terminus of amino acids 105–235 near the zinc finger region [21]. Both antibodies recognize full-length and alternatively spliced human FoxP3. For intracellular staining, T cells were washed, fixed, and permeabilized using the kit of e-Biosciences (Staining Buffer Set no. 00-5523-00) according to the manufacturer’s instructions.

Surface expression of CD25 was analyzed on responder T cells, Treg, and γδ T cells after the cells were stained with anti-CD25 mAb clone 2A3 (BD Biosciences).

Modulation of inhibitory and co-stimulatory molecules on the surface of responder or γδ T cells after co-culture and before and after stimulation with A/E beads was determined by using the following mAb: anti-CD152 (CTLA-4, clone BNI3), anti-CD28 (clone L293), anti-PD1 (clone EH12.2H7), anti-PDL-1 (clone MIH1), anti-CD80 (clone L307.4), and anti-CD86 (clone 2331) (R&D Systems, Minneapolis, MN).

For the phosphorylation analysis of Akt at S473 and T308, NF-κBp65 at S529, extracellular-signal-regulated kinase (ERK) 1/2 at pT202/pY204, p38 MAPK at pT180/pY182, and Stat3 at Y705, we used the modified Phosflow™ Protocol III (BD Biosciences). Briefly, γδ T cells (2 × 10^5^) pre-incubated with medium or a mixture of TLR2L for 22 h followed by a washing step were incubated with 2 μg/mL anti-CD3 (clone OKT3) mAb and with 1 μg/mL anti-CD28 mAb for 30 min on ice. Cells were washed with ice-cold X-VIVO 15 medium, and stimulation was performed by cross-linking of anti-CD3/-CD28 mAb with 10 μg/mL rabbit-anti-mouse (rαm) immunoglobulin Ab for different time periods at 37 °C. Thereafter, cells were fixed with pre-warmed Cytofix™ Buffer (no. 554655; BD Biosciences) at 37 °C for 10 min, washed, permeabilized with Phosflow™ Perm Buffer III (no. 558050; BD Biosciences) on ice for 30 min, washed, and stained with phycoerythrin (PE)-conjugated anti-NF-κBp65 (pS529) clone K10-895.12.50, Alexa Fluor 647-labeled mouse anti-phospho-p38 MAPK (pT180/pY182) clone 36/p38, or anti-phospho-Akt-Ser473 clone M89-61 (all from BD Biosciences). All samples were analyzed on a FACSCalibur flow cytometer (BD Biosciences) using CellQuestPro software.

### Statistical analysis

Student’s two-tailed *t* test (paired data) was used to analyze the statistical significance of differences.

## Results

### γδ T cells suppress expansion of responder T cells

**Fig. 1:**
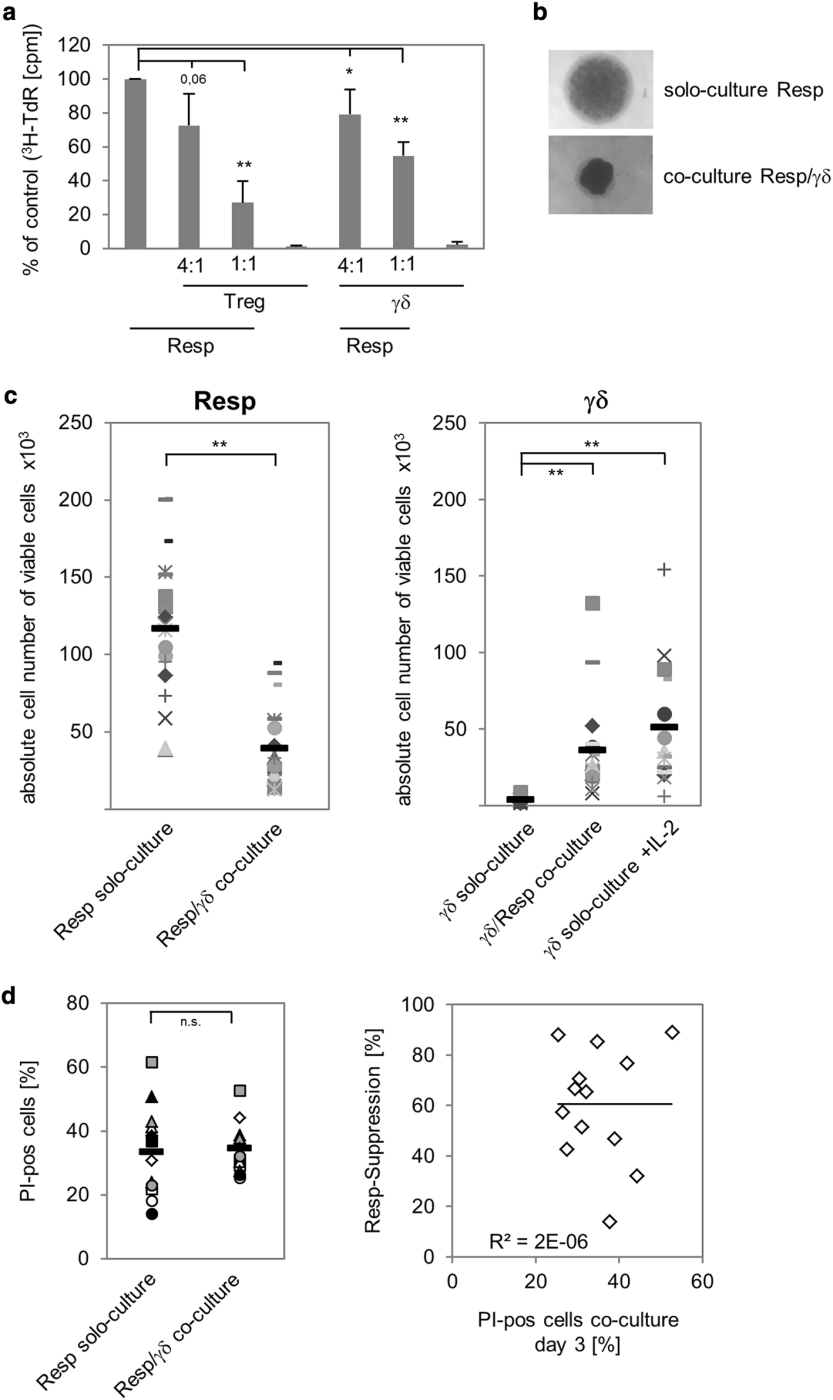
γδ T cells suppress responder T cell (*Resp*) proliferation. 10^4^ negatively isolated Resp (CD4^+^CD25^-^) were co-cultured at the indicated ratio with positively isolated γδ T cells (*γδ*) or regulatory T cells (*Treg*). The cells were stimulated with T cell A/E beads coated with 0.5 μg/mL anti-CD2, 10 μg/mL anti-CD3, and 10 μg/mL anti-CD28. **a** Proliferation was measured by tritiated thymidine (^*3*^ *H-TdR*) incorporation after 3 days of stimulation. Results are shown as relative proliferation in comparison to solo-cultured Resp, whose proliferation was set to 100 %. Results are presented as the mean ± standard deviation (SD) of four independent experiments with triplicate determinations. **b** Microscopic analysis of 7-day cell cultures (magnification ×50). **c** Absolute cell number of viable Resp or γδ T cells in solo- or co-culture (1:1 ratio) was analyzed in 18 different donors by the standard cell dilution assay (SCDA) after 7 days of stimulation. Medium of solo cultivated γδ T cells was supplemented with 50 U interleukin (IL)-2. The mean value of quadruplicate (solo-culture, co-culture) or triplicate (γδ solo-culture + IL-2) determination for each donor is depicted as *one symbol*. *Black bars* Mean values of the different experiments, *asterisks* statistical significance according to Students *t* test (*p ≤ 0.05; **p ≤ 0.01). **d** Cell death was measured 3 days after stimulation in 13 different donors by propidium iodide (*PI*) incorporation into cells. Relative responder suppression was defined as percentage reduction of absolute Resp number in co-culture compared to solo-culture on day 7

Recently, we reported that activated human Vδ2-expressing γδ T cells negatively regulate the proliferative response of αβ T cells against antigens in the presence of IL-12-producing DC or against strong recall antigens or alloantigens in the presence of APC [17]. To examine whether freshly isolated γδ T cells also exert suppressive function on αβ T cells in the absence APC, we used a previously established APC-free suppression assay [43]. This assay is based on the co-culture of magnetically purified CD4^+^CD25^−^ responder T cells with CD4^+^CD25^high^ FoxP3^+^ Treg stimulated with activation/expander T cell beads (A/E beads) in the absence of APC. As illustrated in Fig. 1a, the expansion of responder T cells was significantly, but not completely inhibited upon addition of graded numbers of γδ T cells, similar to the addition of Treg. The proliferation of responder T cell clusters cultured alone (in solo-culture) compared to the co-culture with γδ T cells supported this observation, as shown in Fig. 1b. As expected, γδ T cells as well as Treg did not expand after stimulation with A/E beads in the absence of responder T cells or exogenous IL-2 (Fig. 1a). At the 1:1 ratio, responder T cell proliferation was more potently suppressed by Treg than by γδ T cells (Fig. 1a), possibly due to a reciprocal expansion of γδ T cells in co-culture with stimulated responder T cells producing IL-2. γδ T cells are poor IL-2 producers, and proliferation of γδ T cells in vitro depends on the endogenous IL-2 production of stimulated responder T cells or the exogenous supply of IL-2 [12, 17]. To address whether the suppression of responder T cell proliferation by γδ T cells was also accompanied by a reciprocal expansion of γδ T cells, we determined the absolute cell number of co-cultured responder T cells and γδ T cells. As shown in Fig. 1c, the reduction in the number of responder T cells in co-culture with γδ T cells compared to that of responder T cells in solo-culture (left part Fig. 1c) was accompanied by the simultaneous expansion of γδ T cells in co-culture with responder T cells (right part Fig. 1c). γδ T cell expansion was also analyzed in the presence of exogenous IL-2, under which condition γδ T cells expanded, and in solo-culture without IL-2, where γδ T cells did not proliferate [right part of Fig. 1c; Electronic Supplementary Material (ESM) Fig. 1A]. To exclude the possibility that the γδ T cell-mediated suppression was due to the competition for IL-2, we added 50 U/mL exogenous IL-2 to the suppression assay. Addition of exogenous IL-2 led to enhancement of responder T cell expansion in their solo-culture, but after co-culture with γδ T cells responder T cell proliferation was still suppressed (ESM Fig. 1A). Moreover, trans-well experiments with responder and γδ T cells suggested a contact-dependent mechanism for the suppression because no suppression was observed in the majority of the tested donors after separation of the two T cell populations (ESM Fig. 1B). These results are supported by the observation that freshly isolated γδ T cells did not release suppressive cytokines, such as IL-10 and TGF-β, after stimulation with A/E beads and that the addition of anti-IL-10 or anti-TGF-β Ab did not abolish the γδ T cell-mediated suppression of responder T cells (data not shown).

To examine whether a subpopulation of Vδ2 γδ T cells exerts this suppressive capacity, we separated Vδ2 γδ T cells according to their CD27 expression into subpopulations of CD27-positive cells (naïve, CM) and CD27-negative cells (EM, TEMRA), respectively. In contrast to CD27-negative cells, the majority of the CD27-positive cells co-expressed CD28 (data not shown). Although the suppressive activity of CD27-positive cells was slightly higher than that of CD27-negative cells, we did not observe a significant difference in suppressive function between CD27-negative and CD27-positive cells (data not shown).

In further experiments, we analyzed whether cytotoxic activity of γδ T cells after initial stimulation with A/E beads was responsible for suppression, possibly due to induced cell death of responder T cells. However, we did not observe an increased number of dead cells after co-culturing γδ T cells with responder T cells at the time point when suppression takes place (Fig. 1d). Moreover, suppression of responder T cells co-cultured with γδ T cells was not inhibited by the Fas–Fc fusion protein or neutralizing anti-Fas mAb, nor by pan-caspase inhibitor zVAD-fmk (data not shown). This observation fits well with the results published by Klas et al. [45] showing that resting responder T cells are refractory to cell death for several days. Additionally, TNF-RI-, TNF- BPII-, TRAIL-R1-, or TRAIL-R2-Fc fusion proteins or H_2_O_2_ scavenger catalase had no effect on the abrogation of suppression (data not shown).

Taken together, these results indicated that γδ T cells can suppress responder T cells after activation with A/E beads, also in the absence of APC, but that stimulus with anti-CD28 mAb was necessary to induce the suppressive activity of the γδ T cells (data not shown).

### Characteristic Treg markers are not expressed on γδ T cells

**Fig. 2:**
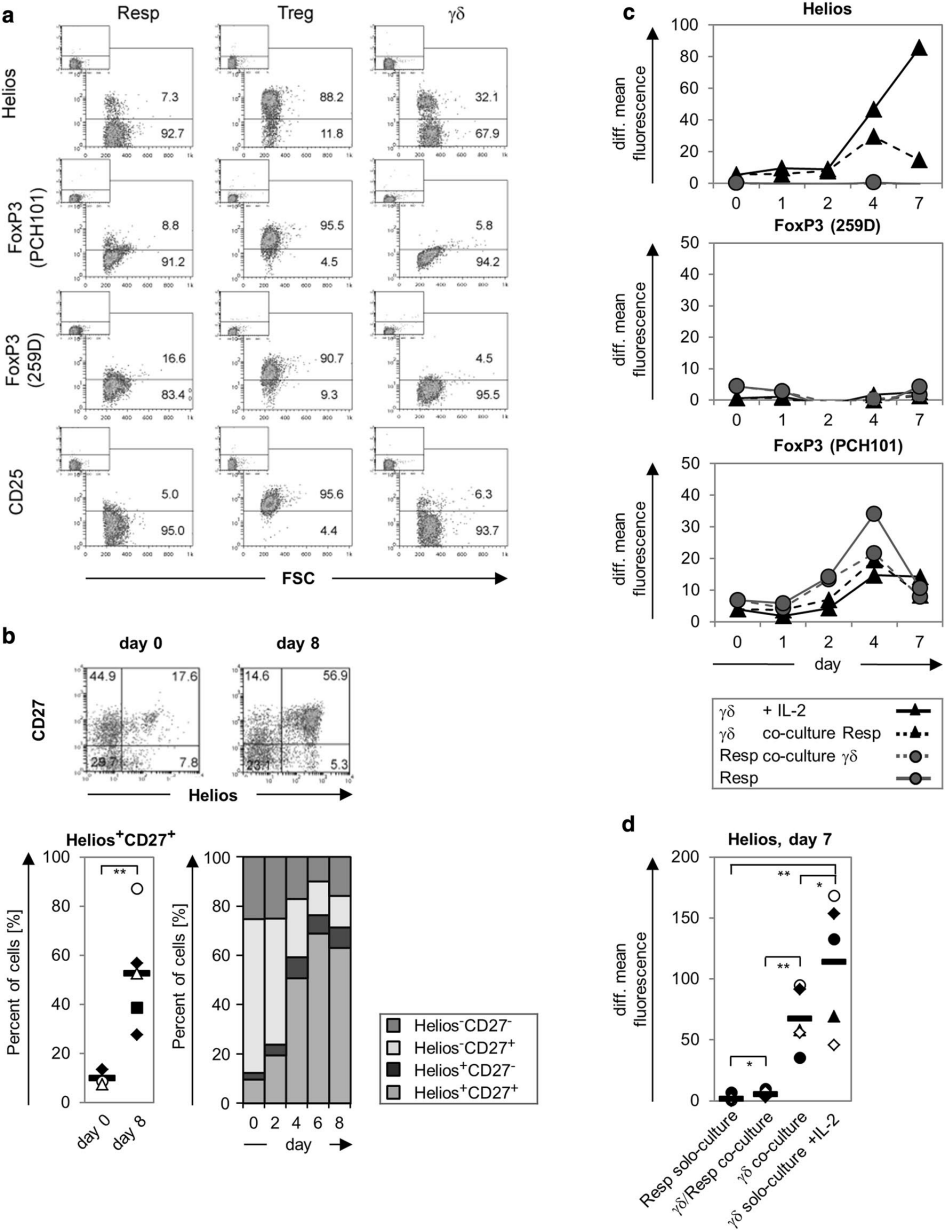
Expression of Treg-associated markers by γδ T cells. **a** 10^5^ purified Resp, Treg, or γδ T cells were stained with the indicated antibodies. The expression of Helios and FoxP3 was determined intracellulary and CD25 expression on the cell surface by flow cytometry. For FoxP3 staining, two different anti-FoxP3 monoclonal antibodies (mAb) were applied, as indicated. The *small-sized dot-blots* located in the *upper left edge* of the *large-sized dot-blots* show the isotype controls. The *numbers* in the *dot-blots* represent the relative proportion. One representative out of five experiments is shown. **b** Co-expression of CD27 and Helios was determined in a time-course study on/in A/E beads-stimulated γδ T cells. Mean values of the relative co-expression from three different donors are depicted in a bar chart. The co-expression on day 0 or 8 after stimulation is depicted for one representative donor in a dot-blot and for five different donors in a scatter plot.** c**, **d**. Changes in the expression of the analyzed transcription factors in solo- or co-cultured T cells after A/E beads-stimulation are displayed in a time-course study (**c**) or 7 days after stimulation (**d**). *Solid lines* Mean values of the mean fluorescence intensity of the indicated γδ- or responder T cells from at least three different donors cultured alone, *dashed lines* co-cultured cells. The mean fluorescence intensity of the appropriate isotype control was subtracted from that of the analyzed transcription factors [= difference (*diff.*) of median fluorescence intensity]

To obtain more insight into the phenotype of suppressive Vδ2 γδ T cells, we analyzed putative Treg markers such as Helios, FoxP3 (with the clone PCH101 or 259D), and CD25 in/on freshly isolated γδ T cells (Fig. 2a). As negative and positive controls, we used freshly isolated responder T cells and freshly isolated Treg, respectively. As expected, responder T cells did not express any of the tested putative Treg markers, while freshly isolated Treg were strongly positive for all markers (Fig. 2a). Intracellular FoxP3 as well as CD25 on the cell surface were not expressed by freshly isolated γδ T cells, while one-third of the γδ T cells expressed intracellular Helios (Fig. 2a, [46]). The percentage of freshly isolated γδ T cells co-expressing CD27 and Helios was very weak at day 0, but it increased after stimulation with A/E beads (Fig. 2b). In subsequent time course experiments over several days, we investigated whether Helios or FoxP3 expression was modulated after the stimulation of γδ T cells, responder T cells, or co-cultured responder/γδ T cells by A/E beads (Fig. 2c, d). Interestingly, the mean fluorescence intensity of intracellular Helios expression was significantly upregulated after γδ T cell stimulation when cells were solo-cultured but upregulated to a lesser extent when the cells were co-cultured with responder T cells. In contrast, responder T cells virtually did not upregulate Helios expression. The determination of the relative percentage revealed that about one-half of the A/E beads-stimulated γδ T cells co-expressed Helios and CD27 and that nearly 90 % of Helios expressing γδ T cells were CD27 positive, which was independent of responder T cells presence (Fig. 2b, c). After stimulation, FoxP3 expression, as detected with the PCH101 mAb, was enhanced under all culture conditions but most efficiently in responder T cells in solo culture. In contrast, when the 259D mAb was used, FoxP3 expression was neither increased in responder T cells nor in γδ T cells (Fig. 2c). We concluded from these data that freshly isolated γδ T cells with a suppressive capacity display a different phenotype from that of nTreg.

### Inhibitory receptor–ligand interactions involved in suppressive functions of γδ T cells

**Fig. 3:**
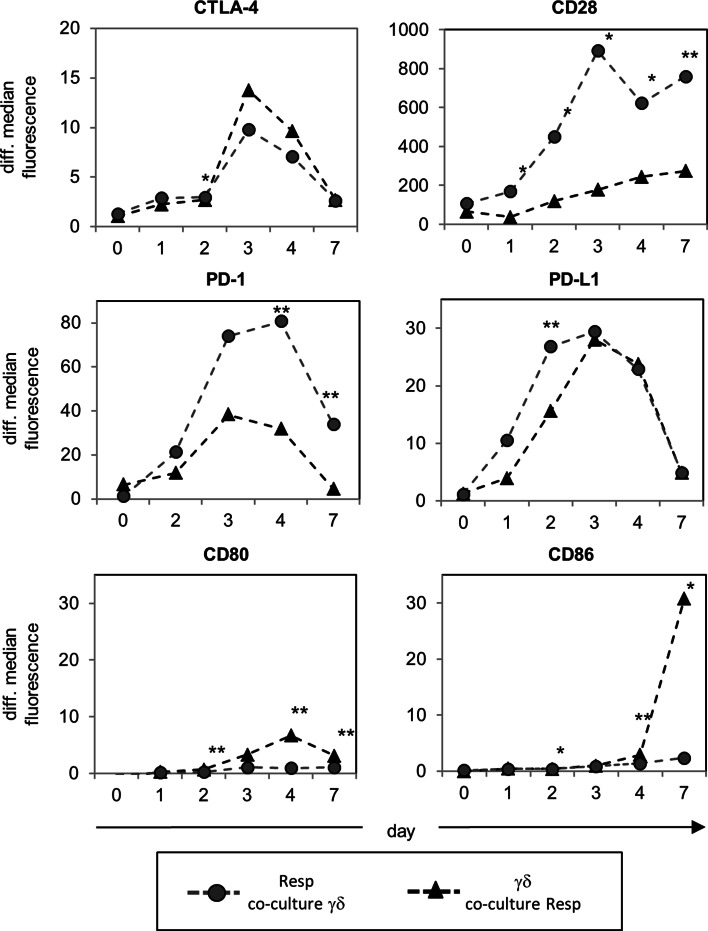
Time course of the expression of inhibitory and co-stimulatory surface molecules on co-cultured γδ T cells and Resp. 10^5^ purified Resp and γδ T cells were co-cultured and stimulated by using T cell A/E beads for the indicated time points. The surface expression of the indicated inhibitory and co-stimulatory molecules was analyzed by flow cytometry in co-cultured Resp (*filled circle*) or in γδ T cells (*filled triangle*) over a time course. *Symbols* Mean values of differential median fluorescence intensity (isotype control was subtracted) of four different donors, *asterisks* statistical significance (*p ≤ 0.05; **p ≤ 0.01)

Cytotoxic T lymphocyte-associated antigen-4 as well as PD-1 and CD28 have been suggested as critical regulators of Treg function [35]. We tested whether the interaction of inhibitory and co-stimulatory cell surface molecules influences the interplay of co-cultured responder- and γδ T cells after activation with A/E beads. We observed that both T cell populations upregulated CTLA-4 and PD-L1, whereas the median fluorescence of CD28 and PD-1 was mainly increased by responder T cells (Fig. 3). CD80 and CD86 mainly expressed by APC were significantly upregulated by γδ T cells 3 days after activation with A/E beads, but not by responder T cells.

**Fig. 4:**
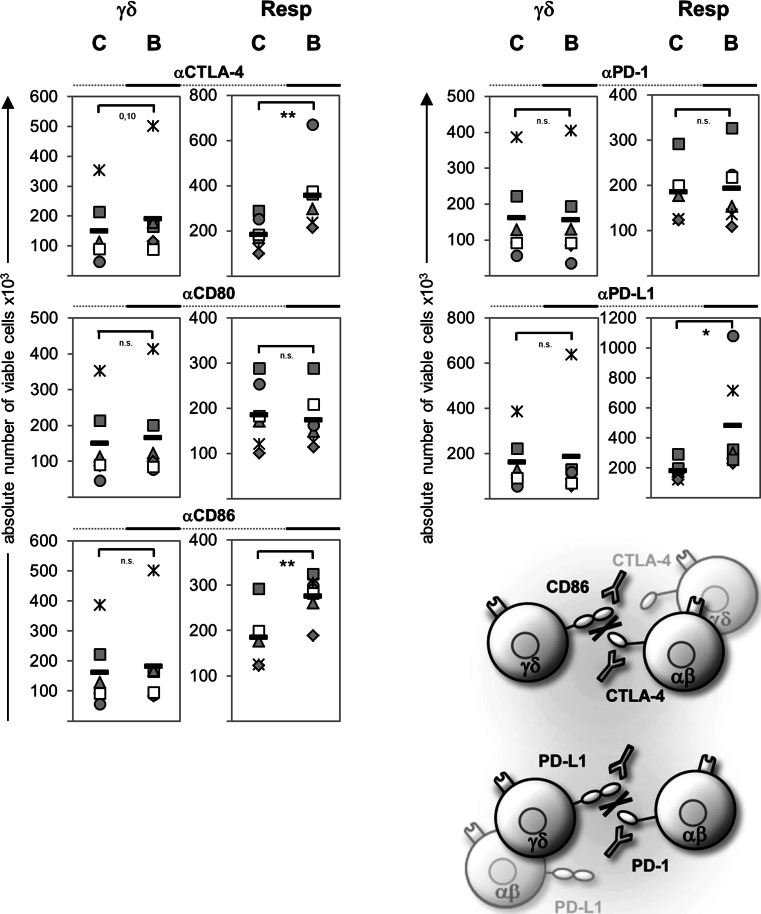
γδ T cell-mediated suppression of Resp is modulated by blocking antibodies. 10^4^ purified γδ T cells were co-cultured with 10^4^ Resp. The T cells were stimulated with plate-bound anti-CD3 (2 μg/mL) and soluble anti-CD28 (1 μg/mL). To examine the influence of the interaction between the surface molecules shown on the presented scheme, we also coated the plates with the indicated blocking antibodies (*B*) or the appropriate isotype controls (*C*) at a concentration of 5 μg/mL. The absolute number of viable γδ T cells (*γδ*) or Resp was determined by the SCDA method on day 6 after stimulation. *Symbol* Data of one donor, *black bars* mean value of at least six different experiments, *asterisks* statistical significance (*p ≤ 0.05; **p ≤ 0.01). *ns* Non-significant

To determine the nature of the suppressive effect, we used blocking antibodies to disrupt the interactions between CTLA-4:CD86, CTLA-4:CD80, or PD-1:PD-L1, respectively. Blocking antibodies had only very weak effects on γδ T cell proliferation in comparison to the appropriate isotype controls. However, γδ T cell-mediated suppression of responder T cell proliferation was abolished by anti-CTLA-4, anti-CD86, or anti-PD-L1 mAb, thereby blocking the CTLA-4 or PD-1 signal, respectively (Fig. 4). These results suggested that CTLA-4 expressing responder T cells could be downregulated by the ligation of CD86 expressed on γδ T cells, while PD–1 expressing responder T cells could be influenced by PD-L1-expressing-γδ T cells as well as by αβ T cells. Anti-CD80 mAb did not affect the interplay between responder T cells and γδ T cells, which can be explained by the weaker expression of CD80 on γδ T cells co-cultured with responder T cells than on those in solo-culture (Fig. 3; data not shown).

### TLR2 ligands partially abrogate the suppressive activity of γδ T cells

**Fig. 5:**
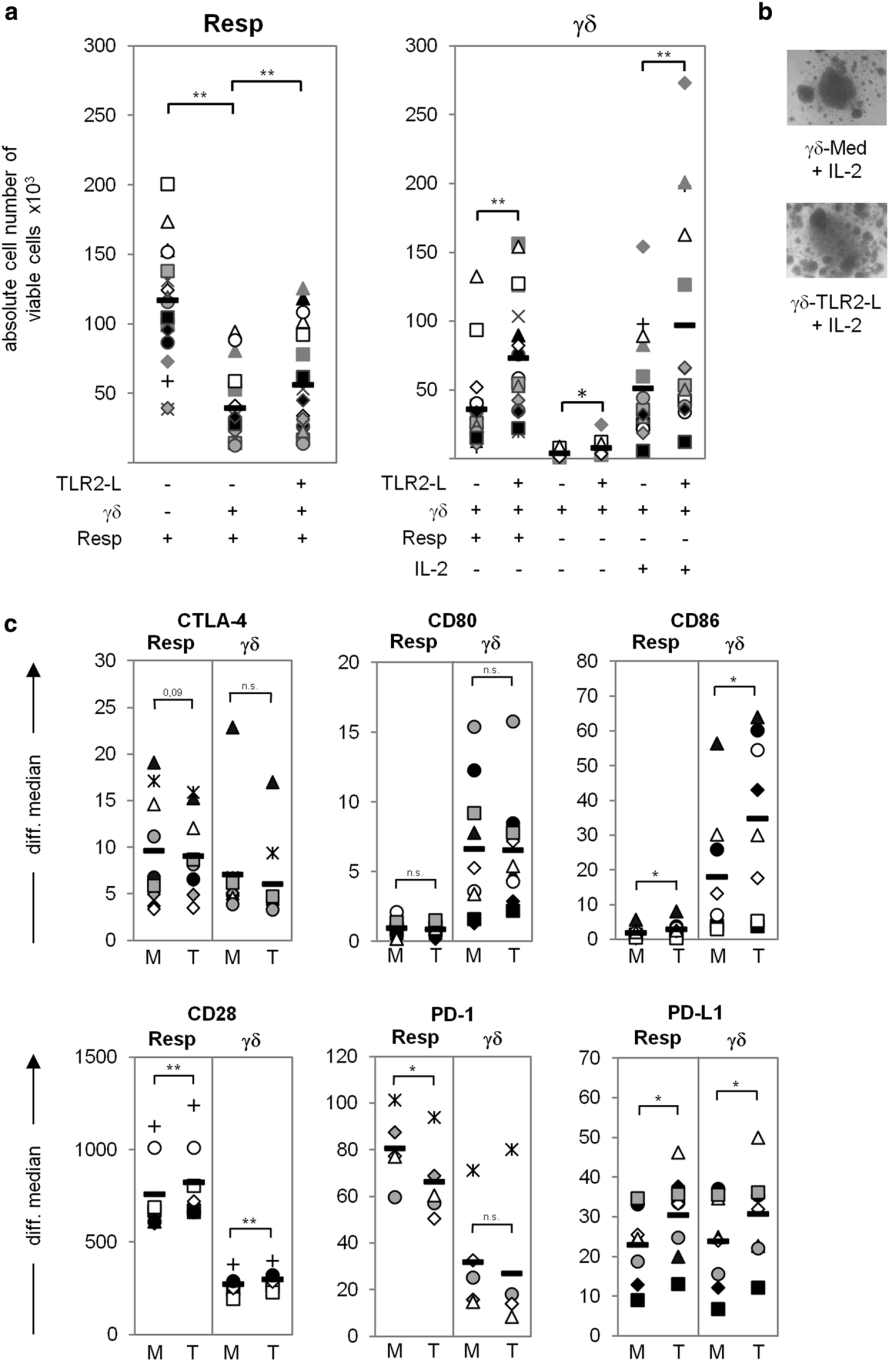
Toll-like receptor 2 ligands (*TLR2-L*) partially abrogate the γδ T cell-mediated suppression of Resp. 10^6^ freshly isolated γδ T cells were pre-incubated in the presence of medium or a TLR2-L mixture (2 μg/mL Pam_2_CSK4, 2 μg/mL Pam_3_CSK4, and 1 μg/mL FSL-1) for 22 h at 37 °C. Thereafter, 10^4^ γδ T cells or Resp were solo- (supplemented with 50 U IL-2 where indicated) or co-cultured. All cells were stimulated with T cell A/E beads. **a** The absolute number of viable Resp or γδ T cells was analyzed by the SCDA method 7 days after stimulation. The mean value of quadruplicate (Resp solo-culture, co-culture) or triplicate (γδ solo-culture + IL-2) determination for each donor is presented as one* symbol*. *Black bars* Mean values of 18 different experiments. **b** In parallel, microscopic inspection of the medium-treated (*γδ-Med*) and TLR2-L-pre-incubated γδ T cells (*γδ-TLR2-L*) (magnification ×50) is shown on day 7 after stimulation. **c** Surface expression of the indicated molecules was analyzed on medium-treated (*M*) or TLR2-L-pre-incubated (*T*) γδ and on co-cultured Resp T cells by flow cytometry at the time point of highest expression. *Symbol* Data of one donor, *black bars* mean value of at least five different experiments, *asterisks* statistical significance (*p ≤ 0.05; **p ≤ 0.01)

Toll-like receptor 2 ligands are able to abrogate Treg-mediated suppression in vitro [39]. The inhibition of responder T cell proliferation was significantly reduced by the pre-treatment of γδ T cells with a mixture of the TLR2 ligands Pam_2_CSK4, Pam_3_CSK4, and FSL-1 (Fig. 5a, responder). All freshly isolated γδ T cells as well as γδ T cell lines express TLR2 on the cell surface, as previously shown by us and other groups [11, 47]. Based on the finding that the pre-treatment of γδ T cells with TLR2 ligands abolished suppressive activity, we analyzed the direct effects of TLR2 ligands on γδ T cells as well as the indirect effects of TLR2 ligand pre-treated γδ T cells on responder T cells.

We determined that pre-treatment of γδ T cells with TLR2 ligands significantly enhanced their proliferative capacity in co-culture with responder T cells or after the addition of exogenous IL-2, but not in solo-culture in the absence of IL-2 (Fig. 5a; γδ T cells). The size and number of proliferation clusters of activated γδ T cells pre-treated with TLR2 ligands compared to untreated cells supported this observation (Fig. 5b). In additional experiments, we demonstrated that the IL-2 production of responder T cells was significantly impaired after co-culture with γδ T cells in the presence of A/E beads. However, pre-treatment of γδ T cells with TLR2 ligands significantly restored the reduced IL-2 production of the co-cultured responder T cells (ESM Fig. 2). In contrast to the reduced IL-2 production measured in the suppression assay, IFN-γ, TNF-α, CCL5 (RANTES), and granzyme B production were not impaired, which might be due to the release of these mediators by γδ T cells. Pre-treatment with TLR2 ligands further significantly increased the production of granzyme B, IFN-γ, TNF-α, and IL-2 and also but not significantly of CCL5 (RANTES). The produced mediators all together could positively influence the proliferation of responder T cells (ESM Fig. 2).

Importantly, the modulation of γδ T cell-mediated suppression following pre-incubation with TLR2 ligands was associated with a modulation of expression of immune-regulating cell surface molecules. The inhibitory receptors CTLA-4 and PD-1 were downregulated on responder T cells, while their expression on treated γδ T cells was nearly the same as that of untreated cells (Fig. 5c). In contrast, the co-stimulatory CD28 molecule was significantly upregulated in both cell populations after co-culturing TLR2 ligand-treated γδ T cells with responder T cells. CD86, which binds to CTLA-4 and to CD28, as well as PD-L1 were significantly up-regulated in both cell populations after TLR2 ligand pre-treatment of γδ T cells (Fig. 5c). Helios expression was increased in pre-treated γδ T cells, whereas CD80 expression was not affected (data not shown).

These results suggested that γδ T cell-mediated suppression affected the proliferation of responder T cells as well as their production of IL-2, whereas the release of IFN-γ, TNF-α, MIP-1α, and RANTES and granzyme B were not affected. Moreover, the addition of TLR2 ligands partially abolished the suppression by γδ T cells by downregulating indirectly inhibitory receptors on responder T cells.

### TLR2 ligands enhance MAP kinases, NF-κB, and Akt phosphorylation

**Fig. 6:**
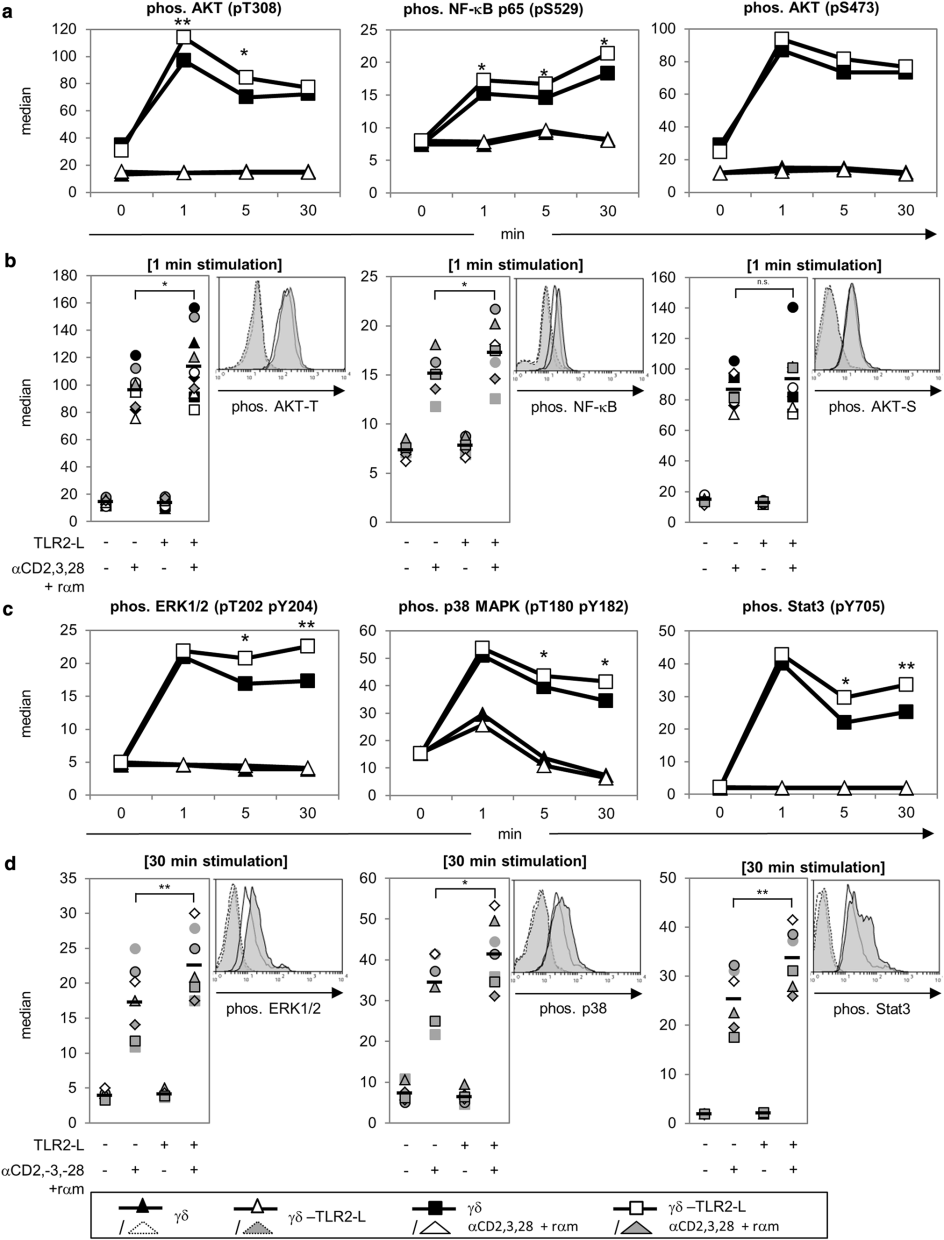
Influence of TLR2-L on the phosphorylation of signaling molecules in γδ T cells. 10^6^ freshly isolated γδ T cells were medium-treated or pre-incubated with a TLR2-L-mixture (2 μg/mL Pam_2_CSK4, 2 μg/mL Pam_3_CSK4, and 1 μg/mL FSL-1) for 22 h at 37 °C. Cells were incubated for 30 min on ice with 2 μg/mL anti-CD3 and 1 μg/mL anti-CD28. The stimulation was started by moving the tubes to 37 °C and the addition of rabbit anti mouse antibody (*rαm*) and stopped at indicated time points by cell fixation followed by permeabilization. Phosphorylated signaling molecules were labeled with specific fluochrome-conjugated antibodies and analyzed by flow cytometry. The median fluorescence intensity of anti-AKT (pT308, pS473), anti-nuclear factor ‘kappa-light-chain-enhancer’ of activated B-cells (*NF-κB/p65*; pS529), anti-extra-signal-regulated kinase (*ERK1/2*; pT202, pY204), anti-p38 (pT180, pY182), and anti-Stat3 (pY705) is depicted. **a**, **c** Mean values of the median fluorescence intensity of at least six donors over a time course of 30 min. **b**, **d** Phosphorylation status at a given time point is presented in a histogram for one representative donor and for statistical analysis in a scatter plot including at least six different donors. *Symbol* Data of one donor, *black bars* mean value for six different experiments, *asterisks* statistical significance (*p ≤ 0.05; **p ≤ 0.01).* MAPK* Mitogen-activated protein kinase

We investigated whether enhanced cytokine production and proliferation after pre-treatment with TLR2 ligands had an influence on several signaling pathways in γδ T cells. TLR2 regulates NF-κB- and MAPK activity and possibly Akt activity, thereby inducing the expression of cytokines and regulating cell survival and proliferation [41, 48]. We investigated the phosphorylation of those molecules shown in Fig. 6 in freshly isolated γδ T cells of several donors in a time course of 1–30 min by a sensitive Phosflow™ method. The time course of mean values from at least six donors is depicted in Fig. 6a, c. The phosphorylation in one donor (histogram) or at least six donors (scatter plot) at the time point of highest discrepancy between the stimulated, differentially pre-treated cells (1 or 30 min) is shown in Fig. 6b, d. Anti-CD3/anti-CD28 mAb activation induced a rapid phosphorylation of Akt at threonine 308 and of NF-κB at serine 529 after 1 min and the effect lasted for >30 min; this activation was enhanced significantly in TLR2 ligand-treated γδ T cells in comparison to untreated γδ T cells. Phosphorylation of Akt at serine 473 was not significantly enhanced in TLR2 ligand-treated γδ T cells (Fig. 6a, b). As a control, unstimulated γδ T cells in medium or with TLR2 ligands were analyzed.

Additionally, we observed that the phosphorylation of ERK1/2, p38, and Stat3 at the indicated phosphorylation sites was induced 1 min after activation with anti-CD3/anti-CD28 mAb, but it was not increased by pre-treatment with TLR2 ligands. However, pre-treatment with TLR2 ligands significantly enhanced phosphorylation after 5–30 min compared to that of untreated γδ T cells (Fig. 6c, d). We obtained comparable results by Western blot analysis. Phosphorylation of ERK2 and p38 was enhanced 1 min after stimulation with anti-CD3/anti-CD28 and was further increased in TLR2 ligand pre-treated γδ T cells 5–30 min after activation in the case of ERK2 and p38 (ESM Fig. 3). Western blot was not as sensitive in detecting the phosphorylation of Akt and NF-κB as the analysis by the Phosflow™ method, although a slightly increased phosphorylation of Akt and NF-κB could be recognized after pre-treatment of γδ T cells with TLR2 ligands compared to untreated cells (ESM Fig. 3).

In subsequent experiments we took advantage of the Phosflow™ method to determine in parallel the phosphorylation of co-cultured responder T cells and γδ T cells. Responder T cells could be discriminated from γδ T cells by combining cell surface staining of the γδ TCR with intracellular anti-phospho Akt or NF-κB mAb. The co-culture of responder T cells with γδ T cells significantly reduced the phosphorylation of Akt (pT308 and pS473) and NF-κB in responder T cells. In co-culture with TLR2 ligand pre-treated γδ T cells the inhibition of Akt and NF-κB phosphorylation was abrogated in responder T cells (ESM Fig. 4, upper panel). TLR2 ligand pre-incubation also enhanced Akt and NF-κB phosphorylation in γδ T cells co-cultured with responder T cells, but only slightly in γδ T cells in solo-culture (ESM Fig. 4, lower panel).

In summary, the enhanced phosphorylation of MAP kinases, NF-κB, and Akt might provide an explanation for the enhanced production of cytokines and an increased proliferation of responder T cells by γδ T cells after TLR2 ligand treatment.

### FoxP3 expression in γδ T cells does not correlate with their suppressive function

**Fig. 7:**
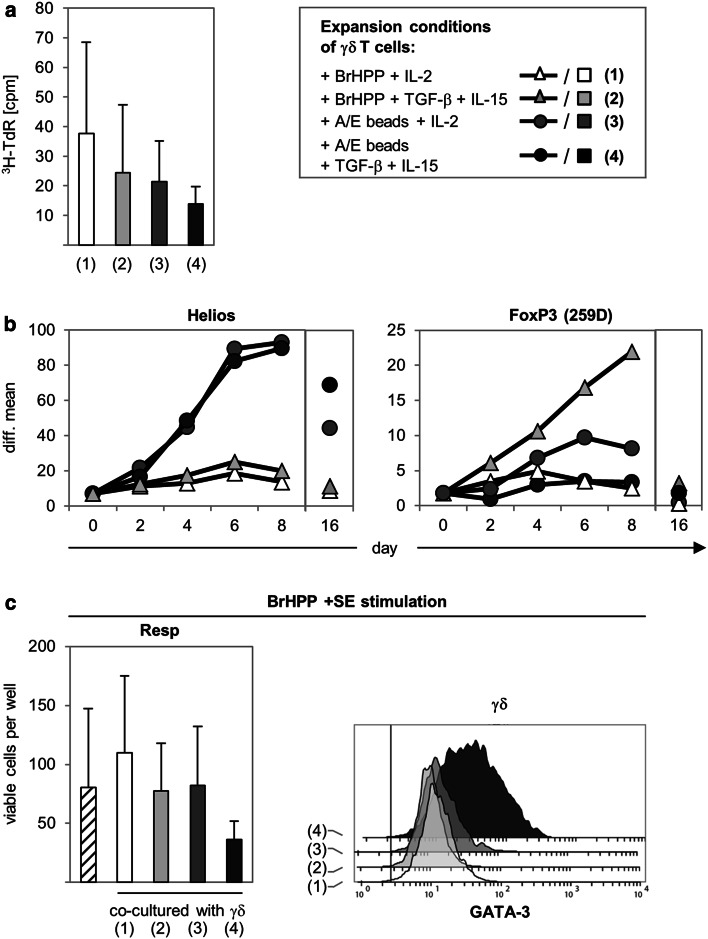
Expression of transcription factors and suppressive function of γδ T cells expanded under different culture conditions. **a**, **b** 5 × 10^4^ freshly isolated γδ T cells were stimulated with either 300 nM phosphoantigen bromohydrin pyrophosphate (*BrHPP*) (in the presence of 2.5 × 10^5^ irradiated peripheral blood mononuclear cells) or A/E beads in the presence of 50 U/mL IL-2 or 1.7 ng/mL transforming growth factor beta (*TGF-β*), 10 ng/mL IL-15, and IL-2 as indicated. **a** Proliferation was determined by ^3^H-TdR incorporation after 5 days of culture. Mean ± SD of five independent experiments with triplicate determination are shown. **b** Intracellular Helios and FoxP3 (259D mAb) expression was analyzed in a time course over 8 days and on day 16. *Symbols* Mean values of difference of median fluorescence intensity (isotype control was subtracted) of seven different donors. **c** 5 × 10^4^ γδ T cells activated under the above-mentioned culture conditions (**a**) were expanded for 16 days after initial stimulation. Thereafter, expanded cells were co-cultured with equal numbers of resting autologous responder T cells (*1–4* in** a**). As a control for suppression, responder T cells were cultured alone (*diagonally lined*). All cell cultures were stimulated with BrHPP, and a mixture of five *Staphylococcus aureus* enterotoxins. Proliferation of responder T cells was analyzed by SCDA 6 days after stimulation. Mean ± SD of five independent experiments with quadruplicate determination are presented. In parallel the transcription factor GATA-3 was determined intracellulary by flow cytometry in these γδ T cells co-cultured with autologous responder T cells

In previous studies using adoptive transfer of γδ T cells as an immunotherapeutic approach, freshly isolated γδ T cells were often expanded under different stimulation conditions (e.g., with PAg BrHPP + IL-2 or anti-CD3/anti-CD28 + IL-2) and, thereafter, cells were re-stimulated before transfer to the patient. We focused on the question of whether BrHPP- or anti-CD3/anti-CD28-activated and -expanded γδ T cells are still able to suppress αβ T cell responses. Moreover, we analyzed if activated and expanded γδ T cells require TGF-β or IL-15 to exert suppressive activity. As shown in Fig. 7a, we observed that freshly isolated, positively selected γδ T cells cultured under the indicated initial stimulation conditions proliferated nearly to the same extent, with γδ T cells activated with BrHPP + IL-2 having a slight advantage, even though the addition of TGF-β seemed to have a negative influence on proliferation independently of the stimulus. Moreover, culture with BrHPP and TGF-β/IL-15 enhanced Th1 cytokine production of γδ T cells (data not shown). The analysis of putative Treg markers on γδ T cells cultured under these different conditions indicated a very prominent expression of Helios in γδ T cells stimulated with A/E beads, whereas BrHPP-activated γδ T cells only slightly expressed Helios. In further experiments, we observed that the anti-CD28 mAb stimulus was responsible for the induction of Helios (ESM Fig. 5). Interestingly, only the γδ T cells cultured with TGF-β and IL-15 expressed FoxP3 (clone 259D), as shown in a time-course study over 8 days (Fig. 7b). FoxP3, but not Helios expression, was transient and decreased in all γδ T cells 16 days after the initial stimulation and before they were co-cultured with responder T cells (Fig. 7b). After re-stimulation of γδ T cells with BrHPP, together with a very strong αβ T cell stimulus (a mixture of five different *S. aureus* enterotoxins), only γδ T cells initially stimulated with A/E beads + TGF-β and IL-15 were still able to suppress the expansion of freshly isolated responder T cells, although FoxP3 was no longer expressed (Fig. 7c). Interestingly, the suppressive activity of these γδ T cells was accompanied by increased GATA-3 expression (Fig. 7c). Moreover, γδ T cells initially stimulated with A/E beads alone still expressed Helios, but were not suppressive after re-stimulation and released more Th1 cytokines than γδ T cells initially stimulated with A/E beads + TGF-β and IL-15 (Fig. 7c; data not shown). In summary, the data suggest that exclusive expression of FoxP3 or Helios in γδ T cells does not correlate with their suppressive function. However, we did observe that the suppressive activity of γδ T cells was influenced through discernible stimulation conditions, suggesting that enhancement of Th1 cytokines in γδ T cells abolishes the suppressive capacity.

## Discussion

Our study has identified specific features of suppressive Vδ2 γδ T cells that differentiate them from nTreg. FoxP3 and Helios expression do not represent specific markers for the suppressive capacity of γδ T cells, but the expression of these molecules seems to be involved in the differentiation of γδ T cells with regulatory function. We also provide insights into the suppressive mechanism of freshly isolated Vδ2 γδ T cells. We demonstrate an important role for the interaction of CD86 on γδ T cells and CTLA-4 on responder T cells which induces a reduced phosphorylation of Akt and NF-κB. Furthermore, we show that the suppressive mechanism of γδ T cells was abrogated by TLR2 ligands, inducing a strong Th1 response and abolishing the γδ T cell-mediated inhibition of Akt and NF-κB phosphorylation in responder T cells (ESM Fig. 4).

Natural Treg, which regulate peripheral tolerance, highly express CTLA-4 and CD25 and can be stained to the same extent with FoxP3 mAb 259D and PCH101 [49]. In our assays, we investigated whether freshly isolated Vδ2 γδ T cells have characteristic features comparable to those of nTreg. We found that similar to nTreg, suppressive Vδ2 γδ T cells required activation, cell–cell contact, and IL-2 to exert their suppressive activity. In contrast to nTreg, however, freshly isolated Vδ2 γδ T cells did not express CTLA-4, CD25, or intracellular FoxP3. After initial activation with A/E beads, cell surface CTLA-4 and CD25 as well as intracellular FoxP3 expression detected with PCH101 mAb but not with 259D mAb was upregulated on Vδ2 γδ T cells, similar to Treg-depleted CD4^+^ responder T cells. The expression of FoxP3 detected with PCH101 mAb but not with 259D mAb has been demonstrated in responder T cells, but it did not coincide with the regulatory function of these cells [21]. In addition, the sensitivity of the 259D mAb and the specificity of PCH101 mAb in natural versus inducible Treg is controversial; FoxP3 expression stained with PCH101 mAb in responder T cells has been found not to correlate with regulatory function [21, 50–52]. We observed FoxP3 expression with the presumably more Treg-specific FoxP3 mAb 259D only in nTreg and in TGF-β-induced γδ T cells, but only weakly in responder T cells or in freshly isolated Vδ2 γδ T cells in a resting as well as in an activated state. Therefore, we conclude that Vδ2 γδ T cells do not possess a characteristic nTreg phenotype. However, initially activated Vδ2 γδ T cells suppressed co-cultured responder T cells, although FoxP3 was not detected by the Treg-specific 259D mAb. Moreover, in accordance with the data of Casetti and colleagues [24], we observed that FoxP3 expression (stained with 259D mAb) could be induced 2 days after the activation of Vδ2 γδ T cells under Treg-inducing conditions (i.e., in the presence of TGF-β and IL-15) and that it increased over a 8-day period. In addition to the data of Casetti et al. [24], we demonstrated that the FoxP3 expression was transient and that it decreased 16 days after the initiation of solo-culture of γδ T cells. Subsequent co-culturing of FoxP3-negative Vδ2 γδ T cells initially expanded with A/E beads + TGF-β, and IL-15/IL-2 still suppressed responder T cell proliferation after re-stimulation (Fig. 7c). These results suggest that the expression of FoxP3 does not correlate closely with the suppressive activity of Vδ2 γδ T cells. Our observation that Vδ2 γδ T cells initially stimulated with A/E beads and TGF-β/IL-15/IL-2 were still suppressive under conditions that induced a strong Th1 response in αβ T cells (after stimulation with five different *S. aureus* enterotoxins) can be due to an upregulation of intracellular GATA-3 in these γδ T cells. The transcription factor GATA-3 controls the production of immunoregulatory cytokines that could specifically counteract a Th1 response. Hansmann et al. [53] demonstrated that human Treg with a memory phenotype often downregulate FoxP3 after in vitro expansion. This downregulation can be accompanied by an upregulation of Th2 genes, such as GATA-3. Caccamo et al. [14] demonstrated that T_CM_ γδ T cells produce less IFN-γ than T_EM_ γδ T cells. We observed that previously FoxP3-expressing Vδ2 γδ T cells, which were still suppressive after initial stimulation with A/E beads and TGF-β/IL-15/IL-2, co-expressed CD27 and Helios, whereas CD27 expression decreased in Helios-negative previously FoxP3-expressing Vδ2 γδ T cells after stimulation with BrHPP and TGF-β/IL-15/IL-2 (Fig. 7b; data not shown). The latter observation could be interpreted as a differentiation of T_EM_ γδ T cells after stimulation with BrHPP and TGF-β/IL-15/IL-2. These data, together with the observation that in freshly isolated A/E beads-stimulated Vδ2 γδ T cells the transcription factor Helios was induced, suggest that CD27/Helios-double positive T_CM_ γδ T cells could be the suppressive subset within the Vδ2 γδ T cells. However, freshly isolated CD27-negative γδ T cells also had a suppressive capacity, although the suppression of freshly isolated CD27-positive γδ T cells was slightly higher compared to that of CD27-negative γδ T cells (data not shown). In this context, conflicting data exist on the function of Helios. Thornton and co-workers [20] described a selective expression of Helios in murine Treg derived from thymic origin, whereas Helios was not expressed in iTreg. However, recently published results demonstrate an upregulation of Helios in murine and human iTreg as well as an association of Helios expression with T cell activation and cell division in non-Treg cells [28–30]. Similar to the results of Akimova and colleagues [28], we observed that <10 % of the freshly isolated human responder T cells expressed Helios. However, a noteworthy upregulation of Helios was not observed in responder T cells after activation with A/E beads in the absence or presence of co-cultured Vδ2 γδ T cells. In contrast to responder T cells, Helios was upregulated in Vδ2 γδ T cells cultured with IL-2 or co-cultured with IL-2-producing responder T cells 2 days after stimulation with A/E beads. Upregulation of Helios expression after the addition of IL-2 has been already described [28]. Our results provide evidence that the upregulation of Helios was due to the stimulation of the Vδ2 γδ T cells with anti-CD28 mAb, which was applied in nearly all experiments in combination with anti-CD3 mAb to activate the Vδ2 γδ T cells. CD28 co-stimulation enhances the stability of IL-2 mRNA and, thereby, the transcription and release of IL-2, which is necessary for T cell expansion as well as for regulatory T cell function, and it can also positively affect Helios expression [33]. Studies by Verhagen and Wraith [54] on the generation of iTreg from non-Treg cells of Rag-deficient Tg4 transgenic mice indicate that a strong co-stimulus given by APC but not by anti-CD28 mAb enhances Helios expression. However, in our experiments with human γδ T cells, the addition of anti-CD28 mAb increased Helios expression in activated γδ T cells, whereas it failed to induce a significant Helios expression in CD4 T cells. CD28 signaling contributes to changes in DNA demethylation at the IL-2 gene locus, and Helios interacts with histone deacetylases and methyl transferases within the nucleosome remodeling and deacetylase (NuRD) complex, which both influences chromatin remodeling that is required for the entry into the cell cycle and differentiation of cells and cell growth [55–58]. In terms of Vδ2 γδ T cells under different culture conditions in our study, we found that Helios expression was more prominent in Vδ2 γδ T cells stimulated with A/E beads (anti-CD2/anti-CD3/anti-CD28) than in those stimulated by PAg BrHPP (in the presence of irradiated PBMC), whereas the proliferative activity was comparable. Therefore, we could not conclude that Helios was induced most prominently in highly dividing Vδ2 γδ T cells, as has been described for αβ T cells [28]. Furthermore, Helios-expressing Vδ2 γδ T cells initially stimulated with A/E beads in the absence of TGF-β and IL-15 did not suppress responder T cells after re-stimulation, suggesting that Helios expression in Vδ2 γδ T cells is not suitable as a marker for suppressive Vδ2 γδ T cells. This finding fits well with the studies of Thornton and co-workers [20] who demonstrated that freshly expanded human iTreg treated with Helios siRNA had normal suppressive activity. From our data, we cannot completely rule out that Helios is involved in the suppressive activity, but we propose that Helios could probably serve more as a marker of differential activation status of Vδ2 γδ T cells than as a Treg-specific marker.

γδ T cells display a broad range of functional plasticity after activation, and Vδ2 γδ T cells can be differentiated on the basis of polarizing cytokines, homing receptors, and lineage-determining transcription factors. Several recent publications have reported that Vδ2 γδ T cells can express an APC-like phenotype after pre-activation for 18–48 h with PAg, autologous B cells, or irradiated HLA-A2-negative Epstein–Barr virus-transformed B cell lines in the presence of IL-2 and IL-15, characterized by an upregulation of APC-specific molecules and APC function [1, 2, 59, 60]. Additionally, we observed that freshly isolated Vδ2 γδ T cells initially stimulated with A/E beads in the presence of IL-2 also upregulated CD86, PDL-1, CD80, and HLA-DR (Fig. 3; data not shown). Moreover, we recognized that initially A/E beads-stimulated—but not (TLR2 ligand) pre-activated—Vδ2 γδ T cells exerted a suppressive capacity. Our observation underlines the functional plasticity of Vδ2-expressing γδ T cells, which appears to be influenced by the activation status, the kind of stimulus of the cells, as well as by the surrounding cytokine milieu. The presence and the strength of co-stimulatory signals appear to be more important for suppression than the expression of specific suppressive markers. Freshly isolated γδ T cells exert their suppressive function only in the presence of anti-CD28 mAb or of APC [17]. CD28 and CTLA-4 are discussed as critical regulators of regulatory T cell homeostasis and function. The dual function of CD28 enables the immune system to efficiently respond against microbes and at the same time to induce regulatory mechanisms required to terminate immune responses [37]. Moreover, our results revealed that CD86, which was upregulated on A/E beads-activated Vδ2 γδ T cells, can bind to CTLA-4, which was upregulated on the activated responder T cells, because the suppressive effect of Vδ2 γδ T cells was abrogated by blocking the CD86:CTLA-4 interaction between Vδ2 γδ T cells and responder T cells. Anti-CD80 did not influence the interaction of Vδ2 γδ T cells and responder T cells, which can be explained by the low CD80 expression on Vδ2 γδ T cells co-culture with responder T cells. We used anti-CD86 and CTLA-4 mAb instead of CTLA-4-Fc to interrupt the CD86:CTLA-4 interaction on the basis of reports that CTLA-4-Fc could also result in reverse signaling through CD86/CD80 [61, 62]. We also examined a possible role of PD-1, which is another member of the CD28 family of receptors with inhibitory functions. PD-1 was upregulated on activated responder T cells to a higher extent than on Vδ2 γδ T cells, and both cell populations expressed the ligand PD-L1, but not PD-L2 (Fig. 3; data not shown). These results suggest the possibility that responder T cells could be regulated by PD-L1 expressed by themselves or by γδ T cells. We observed that the suppressive effect of Vδ2 γδ T cells was abrogated by anti-PD-L1 antibodies, but only slightly by anti-PD-1 antibodies. One explanation for this discrepancy is the possible existence of an unknown second co-stimulatory receptor for PD-L1 in addition to the inhibitory PD-1 [33]. The PD-1/PD-L1 pathway is described as a negative regulator of IFN-γ production, while CTLA-4 inhibits IL-2 synthesis [33]. In our assays IFN-γ production was not decreased, although we cannot exclude the possibility that Vδ2 γδ T cells in the co-culture are the main producer of IFN-γ, whereas IL-2 production is reduced after co-culturing Vδ2 γδ T cells with responder T cells. This possibility emphasizes the role of CTLA-4:CD86 in Vδ2 γδ T cell-mediated suppression on responder T cells.

While initially A/E beads-stimulated γδ T cells were suppressive, pre-treatment with TLR2 ligands partially abolished suppression and led to an enhanced phosphorylation of MAPKs, Akt, and NF-κB resulting in a higher production of Th1 cytokines. We and others have already described that anti-CD3 mAb as well as PAg activation of γδ T cells induce phosphorylation of MAPK ERK2 and of p38 and Akt (at serine 473), which in turn induce the upregulation of crucial target genes, such as IFN-γ and TNF-α [63–65]. Moreover, we observed in our study that combined stimulation with anti-CD3/anti-CD28 mAb induced an additional phosphorylation of Akt at threonine 308 as well as of NF-κB and Stat3 in γδ T cells after stimulation. NF-κB has been described to be involved in the induction of RANTES, whereas Stat3 plays a key role in many cellular processes, such as cell growth [66]. In line with our previous report demonstrating that TLR2 ligands enhance TCR-mediated cytokine and chemokine production of γδ T cells [11], we additionally found that TLR2 ligands significantly increased the activation of MAPKs, NF-κB, Stat3, and Akt at threonine 308. However, the more important question was what happens to the responder T cells in the presence of TLR2 ligand-pre-activated γδ T cells. We observed a downregulation of inhibitory molecules CTLA-4 and PD-1 on responder T cells. Concomitantly, an enhancement of CD28 expression and an increase of effector function of responder T cells resulted in a partial abrogating of the Vδ2 γδ T cell-mediated suppression.

Our results suggest that the pre-activation of γδ T cells could induce APC function of γδ T cells, but not their suppressive capacity. TLR2 ligand-pre-treated γδ T cells and γδ T cells of short-term lines are not able to suppress responder T cells which were activated with a mixture of five *S. aureus* enterotoxins that activate a greater Th1-repertoire of responder T cells than only one *S. aureus* enterotoxin (as applied in our previous study [17]). It is well established that a mixture of *S. aureus* enterotoxins induces a massive cytokine release in responder T cells [67]. We observed that αβ T cells were able to produce high amounts of, for example, IL-6 after combined TCR/TLR stimulation [8]. Enhanced IL-6 production has been described to abrogate Treg-mediated suppression of responder T cells in mice and humans [68, 69]. These results suggest that a bacterial infection (e.g., TLR ligand stimulation) could be a strategy to optimize a Th1-mediated immune response, which results in an abrogation of suppressive capacity of γδ T cells. Only A/E beads, TGF-β, and IL-15-expanded γδ T cells which expressed GATA-3 and Helios in co-culture were able to suppress responder T cell proliferation.

In conclusion, our data provide evidence that Vδ2 γδ T cells have an immunosuppressive potential in the presence of APC [17] or after co-stimulation with anti-CD28 mAb, which could be antagonized by TLR2-ligands or a massive Th1 cytokine production by responder T cells. The ability to control responder T cells by suppressive γδ T cells could have major therapeutic potential for the control of autoimmunity or allergic reactions. Moreover, elimination of suppressive γδ T cells from cells used for adoptive transfer in cancer patients could be useful. 

### Electronic supplementary material

Below is the link to the electronic supplementary material.

**Supplemental Fig. 1** Suppression is not mediated by IL-2 competition, but is cell–cell contact dependent.** a** 10^4^ responder T cells (CD4^+^CD25^-^, Resp) were cultured alone or co-cultured with 10^4^ γδ T cells in the absence or presence of exogenous IL-2 (50 U/mL) for 7 days. After activation with A/E beads, the absolute cell numbers of viable responder T cells and γδ T cells were determined 7 days after culture by SCDA. The mean value of quadruplicate (solo-culture, co-culture) or triplicate (γδ solo-culture + IL-2) measurement for each donor is shown as one symbol. The black bars represent the mean values of 4 different experiments.** b** 10^4^ responder T cells (Resp) were cultured alone or co-cultured with 10^4^ γδ T cells separated from them by the membrane of a trans-well insert. Resp and γδ T cells were stimulated by A/E beads and cultivated for 7 days. Absolute cell numbers of viable responder T cells was determined by SCDA (TIFF 562 kb)

**Supplemental Fig. 2** Influence of TLR2-ligands on the cytokine production. 10^4^ responder T cells were cultured in medium alone or with 10^4^ γδ T cells untreated or pre-treated with a mixture of TLR2 ligands as indicated. After stimulation with A/E beads, supernatants were collected and analyzed by ELISA following the instructions of the manufacturer (R&D Systems; BenderMed System, Vienna, Austria). IFN-γ, TNF-α, and granzyme B production was determined after 72 h and IL-2 or RANTES after 96 h. Each symbol represents one donor, and the bars represent the mean value of different experiments as indicated. Asterisks indicate statistical significance (*p ≤ 0.05 and **p ≤ 0.01) (TIFF 717 kb)

**Supplemental Fig. 3** Phosphorylation of signaling molecules in γδ T cells is enhanced after TLR2-L pre-treatment. 10^6^ γδ T cells were pre-treated in medium or with a mixture of TLR2-L and thereafter stimulated with anti-CD2, anti-CD3, and anti-CD28 mAb cross-linked with 10 µg/mL rαm Ig for the indicated time points.** a** Cells were lysed in NP40 lysis buffer (Fluka Chemie, Buchs Switzerland) with 1% (v/v) of detergent in 20 mM Tris-HCl, 150 mM NaCl with protease inhibitors aprotinin, leupeptin, PMSF, sodium fluoride, and sodium pyrophosphate. Samples were separated on 10% SDS-gel, and protein was transferred to nitrocellulose membranes (Hybond C-Extra, Amersham Biosciences, Braunschweig, Germany). Blots were blocked with 5% BSA and phosphorylated molecules were detected by protein phosphorylation-specific antibodies as indicated. As a loading control, blots were stripped and reprobed with antibodies (as indicated) detecting whole protein levels or with anti-β-actin mAb. Primary Abs were detected by the appropriate HRP-conjugated antibody (Amersham Biosciences, UK). Numbers represent densitometric evaluation.** b** The bars present the values of densitometric evaluation in relation to the corresponding control (TIFF 1099 kb)

**Supplemental Fig. 4** Phosphorylation of signalling molecules in γδ T cells co-cultured with responder T cells. 10^4^ responder T cells were cultured alone or in the presence of freshly isolated γδ T cells pre-treated in medium or with a TLR2-L mixture. After A/E bead stimulation, cells were cultured for 3 days, then fixed and subsequently permeabilized. Indicated phosphorylated signaling molecules were labeled with specific fluochrome-conjugated antibodies and analyzed by flow cytometry. Mean values of the median fluorescence intensity of at least 4 donors is shown. Each symbol represents the data of one donor, and the black bars present the mean value for 4 different experiments. Asterisks indicate statistical significance (*p ≤ 0.05; **p ≤ 0.01).* n.s.* Non-significant) (TIFF 459 kb)

**Supplemental Fig. 5** Helios is induced after co-stimulation with anti-CD28 mAb. Co-expression of CD27 and Helios was stained with the indicated antibodies. The expression was analyzed by flow cytometry on/in γδ T cells after stimulation with anti-CD3 mAb or A/E beads (anti-CD3 and anti-CD28 mAb coated) after 8 days of cell culture (TIFF 300 kb)

## Abbreviations

APC: Antigen presenting cell
BrHPP: Bromohydrin pyrophosphate
CTLA-4: Cytotoxic T lymphocyte-associated antigen-4
ERK: Extracellular-signal-regulated kinase
MAPK: Mitogen-activated protein kinase
NF-κB: Nuclear factor ‘kappa-light-chain-enhancer’ of activated B-cells
PD-1: Programmed death-1
PI3 K: Phosphoinositide 3 kinase
Stat3: Signal transducer and activator of transcription 3
TLR: Toll-like receptor
