# Supplementary figures and images for: Phenotype and regulation of immunosuppressive Vδ2-expressing γδ T cells
Source: Cell Mol Life Sci. 2013 Oct 4;71(10):1943–60. doi: 10.1007/s00018-013-1467-1 (PMC3997799; doi:10.1007/s00018-013-1467-1)

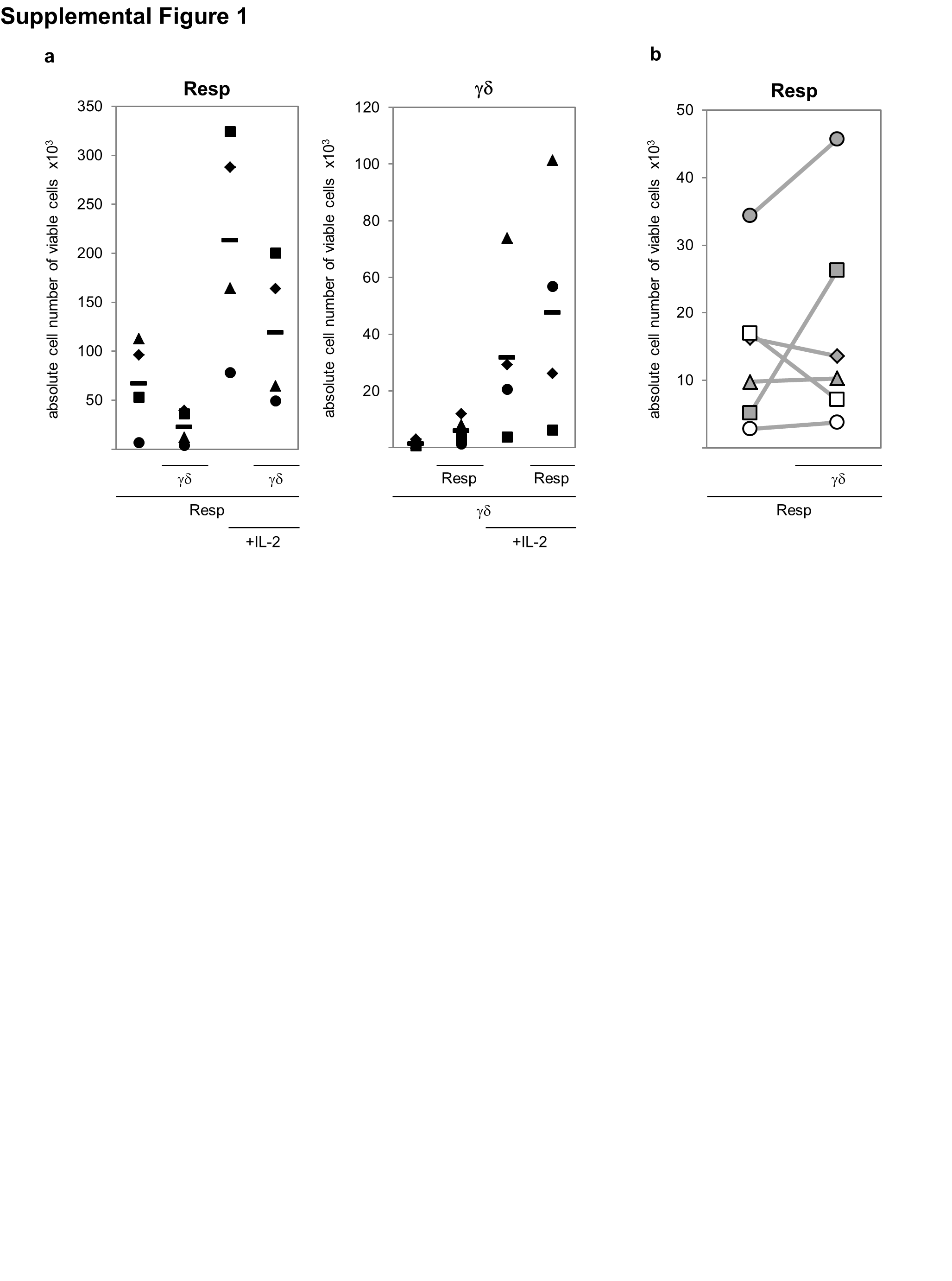

Supplement: Supplementary file 1 — Supplemental Fig. 1 Suppression is not mediated by IL-2 competition, but is cell–cell contact dependent. a 104 responder T cells (CD4+CD25-, Resp) were cultured alone or co-cultured with 104 γδ T cells in the absence or presence of exogenous IL-2 (50 U/mL) for 7 days. After activation with A/E beads, the absolute cell numbers of viable responder T cells and γδ T cells were determined 7 days after culture by SCDA. The mean value of quadruplicate (solo-culture, co-culture) or triplicate (γδ solo-culture + IL-2) measurement for each donor is shown as one symbol. The black bars represent the mean values of 4 different experiments. b 104 responder T cells (Resp) were cultured alone or co-cultured with 104 γδ T cells separated from them by the membrane of a trans-well insert. Resp and γδ T cells were stimulated by A/E beads and cultivated for 7 days. Absolute cell numbers of viable responder T cells was determined by SCDA (TIFF 562 kb) [file 18_2013_1467_MOESM1_ESM.tif]

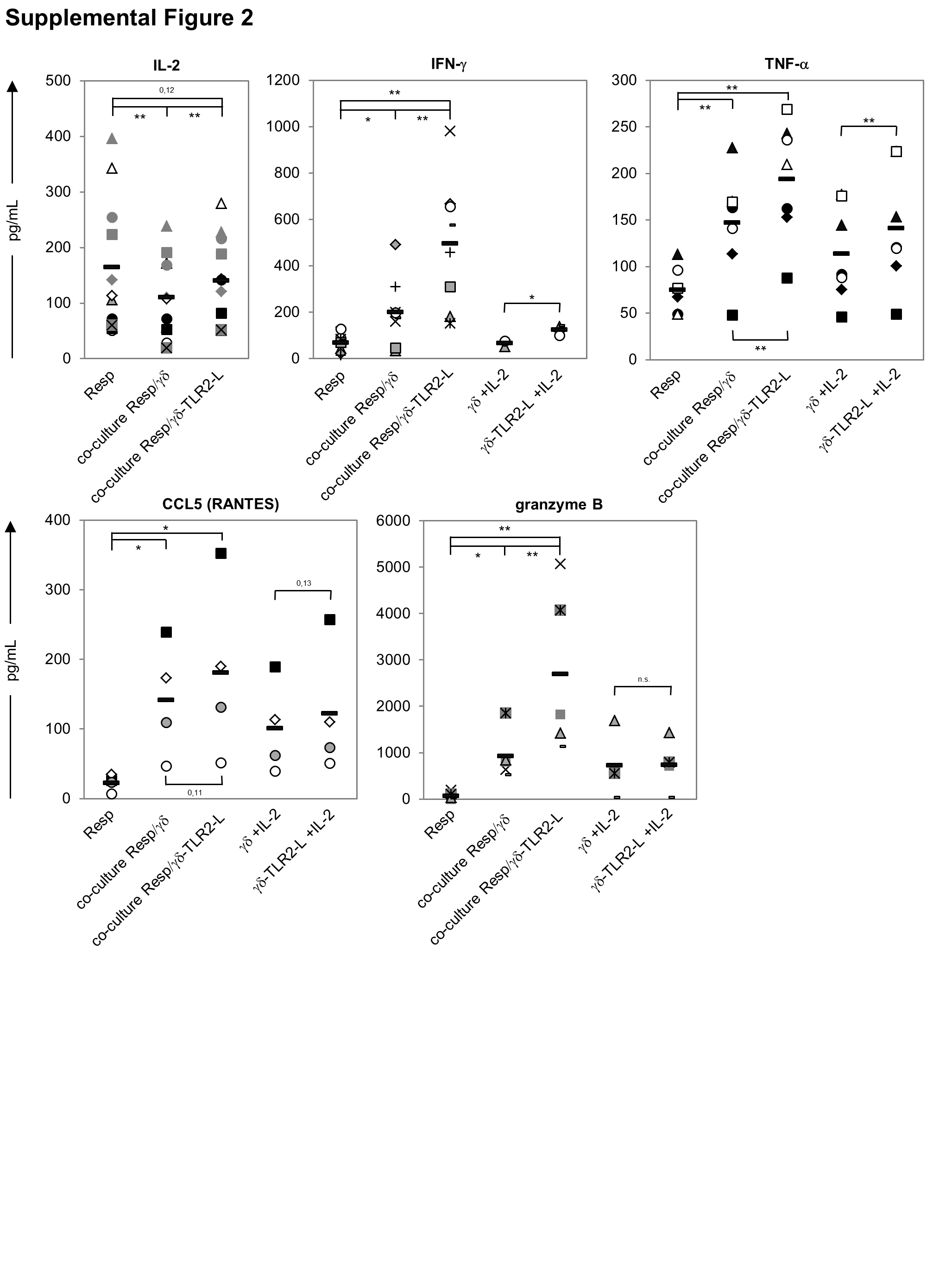

Supplement: Supplementary file 2 — Supplemental Fig. 2 Influence of TLR2-ligands on the cytokine production. 104 responder T cells were cultured in medium alone or with 104 γδ T cells untreated or pre-treated with a mixture of TLR2 ligands as indicated. After stimulation with A/E beads, supernatants were collected and analyzed by ELISA following the instructions of the manufacturer (R&D Systems; BenderMed System, Vienna, Austria). IFN-γ, TNF-α, and granzyme B production was determined after 72 h and IL-2 or RANTES after 96 h. Each symbol represents one donor, and the bars represent the mean value of different experiments as indicated. Asterisks indicate statistical significance (*p ≤ 0.05 and **p ≤ 0.01) (TIFF 717 kb) [file 18_2013_1467_MOESM2_ESM.tif]

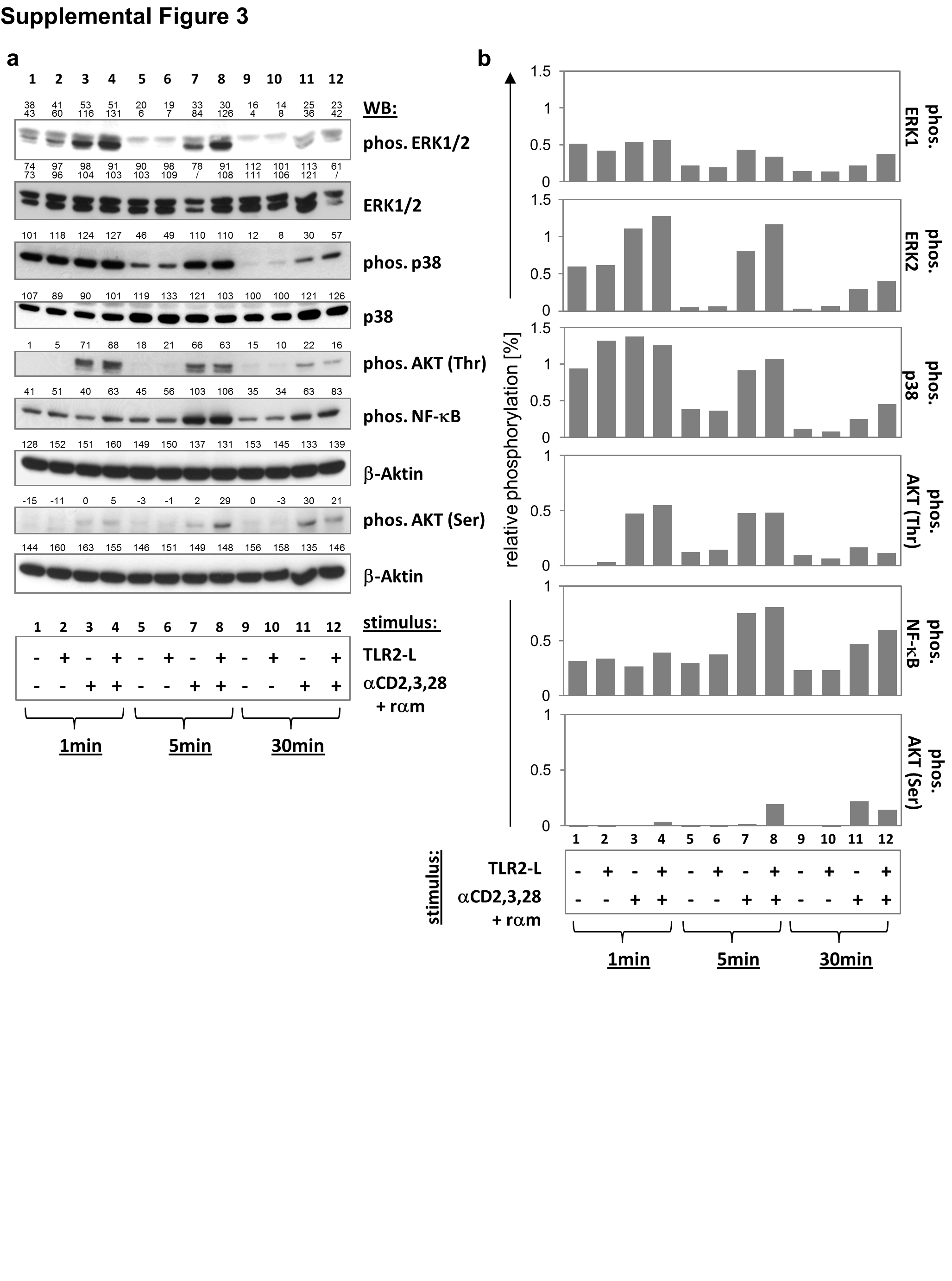

Supplement: Supplementary file 3 — Supplemental Fig. 3 Phosphorylation of signaling molecules in γδ T cells is enhanced after TLR2-L pre-treatment. 106 γδ T cells were pre-treated in medium or with a mixture of TLR2-L and thereafter stimulated with anti-CD2, anti-CD3, and anti-CD28 mAb cross-linked with 10 µg/mL rαm Ig for the indicated time points. a Cells were lysed in NP40 lysis buffer (Fluka Chemie, Buchs Switzerland) with 1% (v/v) of detergent in 20 mM Tris-HCl, 150 mM NaCl with protease inhibitors aprotinin, leupeptin, PMSF, sodium fluoride, and sodium pyrophosphate. Samples were separated on 10% SDS-gel, and protein was transferred to nitrocellulose membranes (Hybond C-Extra, Amersham Biosciences, Braunschweig, Germany). Blots were blocked with 5% BSA and phosphorylated molecules were detected by protein phosphorylation-specific antibodies as indicated. As a loading control, blots were stripped and reprobed with antibodies (as indicated) detecting whole protein levels or with anti-β-actin mAb. Primary Abs were detected by the appropriate HRP-conjugated antibody (Amersham Biosciences, UK). Numbers represent densitometric evaluation. b The bars present the values of densitometric evaluation in relation to the corresponding control (TIFF 1099 kb) [file 18_2013_1467_MOESM3_ESM.tif]

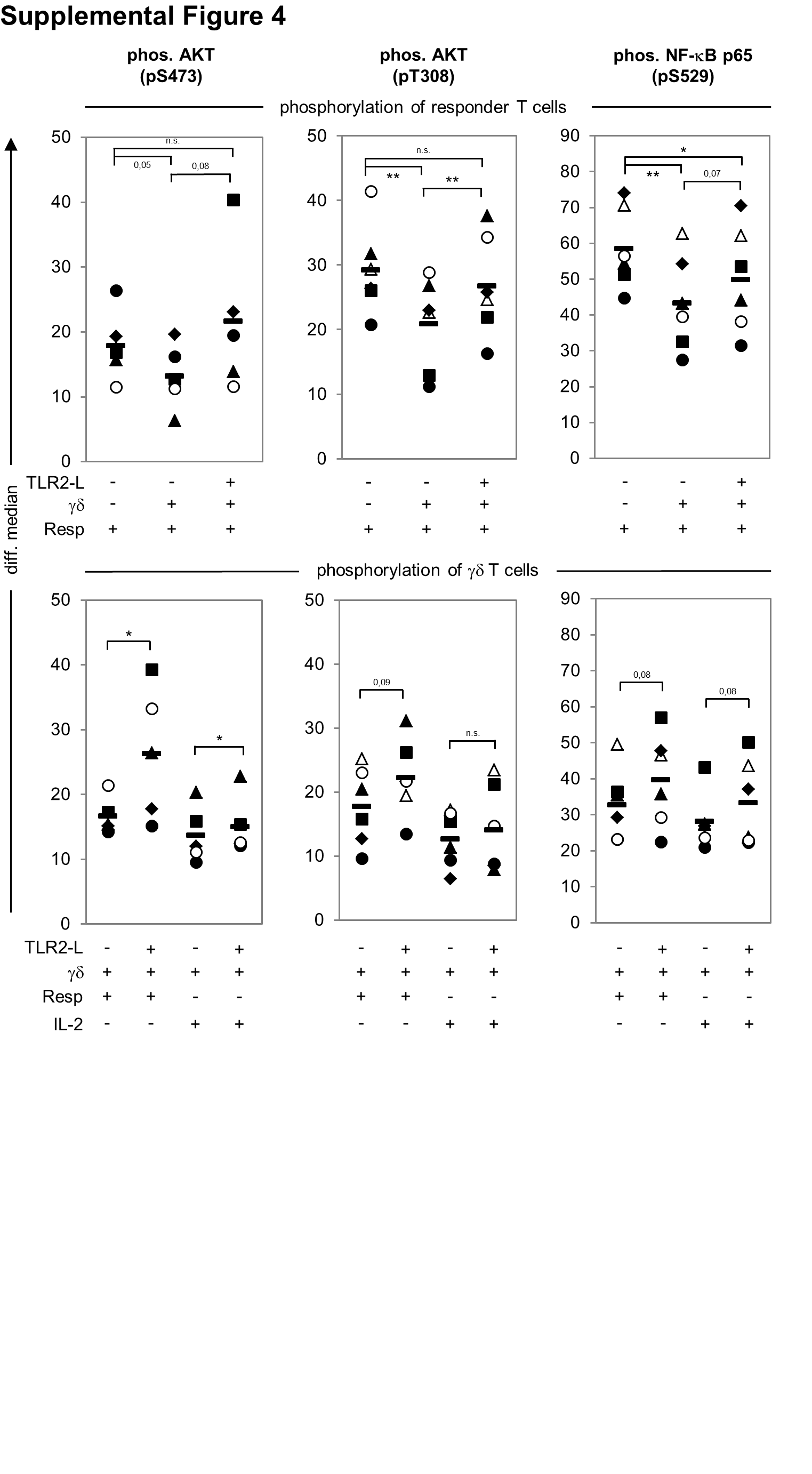

Supplement: Supplementary file 4 — Supplemental Fig. 4 Phosphorylation of signalling molecules in γδ T cells co-cultured with responder T cells. 104 responder T cells were cultured alone or in the presence of freshly isolated γδ T cells pre-treated in medium or with a TLR2-L mixture. After A/E bead stimulation, cells were cultured for 3 days, then fixed and subsequently permeabilized. Indicated phosphorylated signaling molecules were labeled with specific fluochrome-conjugated antibodies and analyzed by flow cytometry. Mean values of the median fluorescence intensity of at least 4 donors is shown. Each symbol represents the data of one donor, and the black bars present the mean value for 4 different experiments. Asterisks indicate statistical significance (*p ≤ 0.05; **p ≤ 0.01). n.s. Non-significant) (TIFF 459 kb) [file 18_2013_1467_MOESM4_ESM.tif]

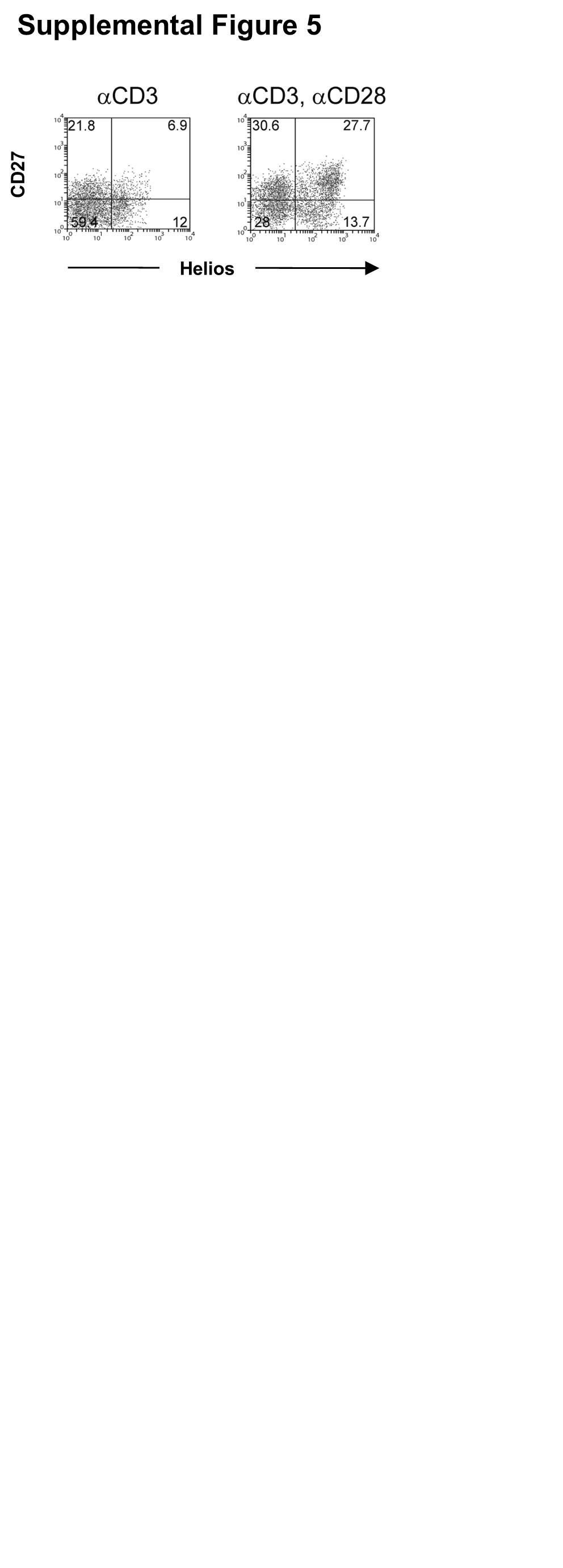

Supplement: Supplementary file 5 — Supplemental Fig. 5 Helios is induced after co-stimulation with anti-CD28 mAb. Co-expression of CD27 and Helios was stained with the indicated antibodies. The expression was analyzed by flow cytometry on/in γδ T cells after stimulation with anti-CD3 mAb or A/E beads (anti-CD3 and anti-CD28 mAb coated) after 8 days of cell culture (TIFF 300 kb) [file 18_2013_1467_MOESM5_ESM.tif]
